# An Overview of Astrocyte Responses in Genetically Induced Alzheimer’s Disease Mouse Models

**DOI:** 10.3390/cells9112415

**Published:** 2020-11-04

**Authors:** Fokion Spanos, Shane A. Liddelow

**Affiliations:** 1 Neuroscience Institute, NYU Grossman School of Medicine, New York, NY 10016, USA; fokion.spanos@student.uva.nl; 2 Department of Neuroscience and Physiology, NYU Grossman School of Medicine, New York, NY 10016, USA; 3 Department of Ophthalmology, NYU Grossman School of Medicine, New York, NY 10016, USA

**Keywords:** Alzheimer’s disease, astrocytes, reactive astrocyte, GFAP, mouse models, APP, Tau, ApoE, TREM2

## Abstract

Alzheimer’s disease (AD) is the most common form of dementia. Despite many years of intense research, there is currently still no effective treatment. Multiple cell types contribute to disease pathogenesis, with an increasing body of data pointing to the active participation of astrocytes. Astrocytes play a pivotal role in the physiology and metabolic functions of neurons and other cells in the central nervous system. Because of their interactions with other cell types, astrocyte functions must be understood in their biologic context, thus many studies have used mouse models, of which there are over 190 available for AD research. However, none appear able to fully recapitulate the many functional changes in astrocytes reported in human AD brains. Our review summarizes the observations of astrocyte biology noted in mouse models of familial and sporadic AD. The limitations of AD mouse models will be discussed and current attempts to overcome these disadvantages will be described. With increasing understanding of the non-neuronal contributions to disease, the development of new methods and models will provide further insights and address important questions regarding the roles of astrocytes and other non-neuronal cells in AD pathophysiology. The next decade will prove to be full of exciting opportunities to address this devastating disease.

## 1. Introduction

Alzheimer’s disease (AD) is the most prevalent neurodegenerative disorder in adults, which manifests clinically through memory impairment and reduced cognition [1,2,3]. Even though it is a disease commonly associated with aging, the primary pathological features of disease, extracellular amyloid beta (Aβ) plaques, and intracellular neurofibrillary tangles (NFTs) of Tau may be present twenty to thirty years prior to clinical symptoms of the disease being noticed [4]. More than ninety percent of AD cases are sporadic, of late onset (late-onset Alzheimer’s disease—LOAD), and of unknown etiology. A minority are of autosomal dominant inheritance carrying mutations in genes that encode disease-associated proteins, such as the amyloid precursor protein (APP) and the presenilins (PSENs) 1 and 2, which cleave APP to produce Aβ [5]. Analyses of families carrying these genes have allowed the formation of the major hypothesis that has driven AD research for the last twenty-five years. There is no evidence supporting a role for *APP* or *PSEN* genes in LOAD, but genome-wide association studies (GWAS) have identified almost 30 genes that make a statistically significant contribution to the genetic risk for the disease, with the *APOE* locus having the greatest impact.

Despite the high number of studies in human AD patients and animal models supporting a major role for Aβ in disease development, there are no agents currently approved by the FDA for use in humans that are designed to inhibit Aβ production, its aggregation, or to enhance its clearance from the brain, and which effectively treat AD [6,7]. This is most likely due to the high complexity of the disease, since several factors involving multiple genetic loci, cellular pathways, and environmental factors contribute to AD development [8]. Experimentally addressing each component individually followed by integrating the spatial, temporal, and kinetic interaction(s) among the different factors is a Herculean task. Since synaptic loss is a major feature of AD, the bulk of cellular studies have focused on primary events in AD neurons either in vivo or in cell culture after exposure to Aβ or Tau aggregates. It has been recently appreciated that the other brain cell types—namely astrocytes, microglia, vascular endothelial cells, and pericytes—play a significant role throughout AD pathogenesis [9,10,11,12].

**Figure 1:**
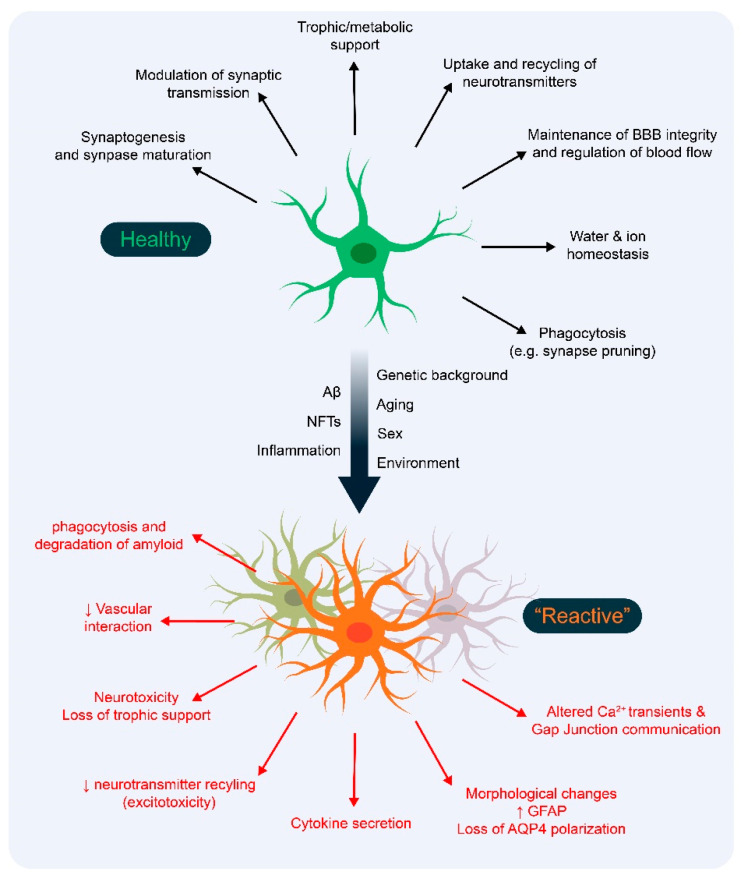
Summary of some main physiological functions of healthy astrocytes (top) and AD-induced changes in reactive astrocytes (bottom, multiple heterogeneous sub-states). Multiple AD-implicated risk factors (indicated next to the black arrow) contribute to changes in astrocyte function. The term “reactive” is representative of multiple reactive astrocyte sub-states that exist, each with differential functions, which are either beneficial or detrimental during AD pathogenesis. These sub-states may change according to disease progression, sex, aging, and underlying mutations or secondary pathology (e.g., inflammation). Abbreviations: Aβ = amyloid beta; AD = Alzheimer’s disease; AQP4 = aquaporin 4 water channel; BBB = Blood-brain barrier, GFAP = glial fibrillary acidic protein; NFT = neurofibrillary tangles (of Tau).

Astrocytes are at least as numerous as neurons in the human brain [13], although their numbers are reported to account for between 20 and 60% of brain cells, depending on the species and brain region [14,15,16]. Their physiological functions include metabolic support of neurons; maintenance of the integrity of the blood–brain barrier (BBB); and regulation of blood flow, synapse formation, and synaptic transmission (Figure 1) [17,18,19,20,21]. Changes in these important astrocyte functions occur in response to injury, infection, and chronic neurodegenerative disorders [22]. The astrocytic response during central nervous system (CNS) damage or disease, termed “astrogliosis” or astrocyte “reactivity”, was first visualized using acidic dyes, and once antibodies had been produced by staining for glial fibrillary acidic protein (GFAP), an intermediate filament marker in astrocytes [23,24]. In humans, astrocyte reactivity is characterized by morphological changes (hypertrophy) and upregulation of GFAP, as evidenced by the increased GFAP-stained volume and branching per astrocyte. Similar changes in GFAP levels occur in mouse models of neurodegenerative diseases [25,26,27,28]. As molecular, biological, and transcriptomic analysis tools became more readily available, gene expression profiles have been used to characterize the astrocytic response. Early studies suggested that astrocyte reactivity was limited to a small number of distinct states dependent on the mode of the initiating injury [29,30]. However, this binary segregation of astrocyte function is controversial and astrocytes in different AD mouse models only partially resemble the complex heterogeneous reactive “sub-states” now reported in complex diseases such as AD [31,32,33]. In any case, various reactive astrocyte sub-states have been described surrounding amyloid plaques in mouse models of AD [34], and more recently in a number of single-cell or nuclei experiments in humans and mice [35,36,37]. These single-cell studies highlight the high degree of transcriptional heterogeneity, but due to a lack of in vitro models for individual reactive astrocyte sub-states, these transcriptomic discoveries have not yet been correlated with potential functional heterogeneity [23,38].

To gain a better understanding of the cellular behavior and molecular response that occur during AD, a large variety of mouse models have been generated (https://www.alzforum.org/research-models/alzheimers-disease). These models shed light on astrocyte contributions to AD pathogenesis and pathology. Neurotoxicity, disrupted aquaporin 4 (AQP4) polarization, and altered Ca^2+^ signaling are some of the ways AD-associated gene mutations impede normal astrocyte function, which in turn are proposed to negatively impact neuronal health and function [30,39,40]. However, rodents do not naturally develop AD, and insertion of several mutations of human genes into mice are required to recapitulate some of the pathological hallmarks present in human patients (i.e., Aβ plaques, neurofibrillary tangles, broadly defined “gliosis”, neuronal death, synapse loss, electrophysiological changes, and cognitive impairment). This has been the subject of many reviews that have concluded that mice fail to recapitulate many aspects of human disease, highlighting a lack of model validity as the main cause of the lack of translatability of new model-based treatments to human patients [41,42,43].

One possible reason for limited mouse-to-human translatability is that the models, while modeling neuron pathology, do not fully recapitulate the glial and immune components of the human disease—likely due to a lack of markers and tools that can be used to understand how these cells change in humans. While non-neuronal cells have received some focus historically, the advent of new markers, specific genetic tools, and use of high-throughput transcriptomics has seen them gain more attention as possible active contributors to neurodegenerative disease. For instance, the involvement of neuroinflammation and vascular dysfunction as significant contributors to the development of AD is now widely accepted [44,45]. However, even when different aspects of AD involving multiple cell types are integrated and studied in mouse models, other factors can also affect disease progression. The genetic predisposition (in humans) or background (in mice) greatly influences AD pathogenesis [46,47]. For that reason, recent outbred and wild-derived AD mouse models have been created, which capture the human AD phenotypic heterogeneity and are believed to improve translatability and allow for personalized medicine. Additionally, cohort studies in mice where the influence of different genetic backgrounds and environmental factors (i.e., diet) are measured are helping to better understand how AD manifests, progresses, and can be treated [48]. The advent and wide availability of new methods and technologies has also contributed to our understanding of AD. Specifically, transcriptomics has allowed for high-throughput genetic sequencing at the cellular level, allowing development of various tools that can be used to better analyze the generated data and compare them across species [49,50,51]. One example includes generating a human co-expression network from AD and non-demented individuals. Then, the RNA sequencing data from one or multiple mouse models can be used to identify differentially expressed genes between transgenic and wild-type mice [49,51]. These genes can be overlapped with human genes in the co-expression network to identify which aspects of AD pathology a specific model recapitulates (i.e., cellular mechanisms, cell types involved). With over 190 genetically induced AD mouse models available, it is becoming difficult to keep track of what is already known about astrocytes in AD, whether the findings are comparable between studies, and what should be addressed next. It is also critical to consider the differences between mouse and human biology and between transgenic models and human AD brains. Do astrocytes respond and behave similarly in humans with AD and in mouse models of AD? Does the endogenous astrocyte population differ between age-comparable naïve humans and mice? Does aging, the highest risk factor for developing AD, differentially contribute to disease progression similarly in humans and mice? What are the advantages and limitations of current AD mouse models? Can these models be improved to increase their translatability? Our review will address these questions in the context of summarizing the main in vivo findings on astrocytes across AD mouse models (see supplementary information regarding literature search) and comparing them with what is known from studies in human AD brains.

## 2. Astrocyte Responses in Genetically Induced AD Mouse Models

The first attempts to model AD pathology in mice took advantage of overexpression of mutated human genes involved in familial AD (FAD). Most of these transgenic (Tg) lines recapitulate some, but not all, aspects of AD pathology. For instance, models with mutations in the *APP* and *PSEN* genes tend to develop extracellular Aβ plaques but in multiple models there is no neuronal loss (e.g., APPswe/PSEN1dE9, APP^NL-G-F^) [52,53]. While Tau may be phosphorylated in some of these models (e.g., APP23 [54]), as a rule there is no evident formation of Tau tangles, even though a number of Tau AD models, but not all, display substantial neuronal loss (e.g., rTg4510, PS19) [52,53]. Human Tau and its mutant forms have also been overexpressed in mice. Again, while these mice may develop tangles, they do not show other features of AD pathology such as Aβ plaques, but several do develop neuronal loss [55]. As a response to this apparent experimental fragmentation, APP, PSEN, and Tau transgenes have been combined to form unique experimental strains [56,57]. Using different cell-type-specific promoters, the transgenes can be expressed in different cells (e.g., *Thy1* promoters for neurons, *Gfap* for astrocytes [58]), allowing for a better mechanistic approach to the understanding of how expression of AD-associated genes affects each cell type, and as a result determination of their contribution to AD pathogenesis. To overcome any issues caused by transgene overexpression, which were common in the early AD models, various knock-in (KI) models, such as APP^NL-F^ (knock-in of humanized *APP* sequence introns 15–17 containing the Swedish and Beyreuther or Iberian mutations [59]), have also been generated. Another major concern involves the type of AD being modeled (familial or sporadic). Most mouse models mimic features of FAD cases, which constitute a relatively small portion of the total number of patients, under the assumption that all or some elements of pathogenesis would be shared with the more common sporadic or LOAD, since the major pathologic features of both are the Aβ plaque and neurofibrillary tangles.

Genetic studies in LOAD patients showed that apolipoprotein-E (*APOE*) is a major genetic risk factor for developing LOAD and individuals carrying the ε4 allele (*APOE4*) are more likely to develop the disease and at an earlier age [60]. The observation made with a traditional genetic approach has been reproduced many times in multiple GWAS, which have also identified almost thirty additional independent loci that are associated with susceptibility to LOAD across different human populations [61,62,63]. The relevance of studying astrocytes in the context of transgenic models of human AD is apparent in the context of the GWAS analyses, which have defined several AD-risk genes, the expression of which are enriched in astrocytes in the CNS (e.g., *APOE*, *CLU*, *SERPINA1*, *CXCL16*) [64,65].

### 2.1. Familial Alzheimer’s Disease Mouse Models

#### 2.1.1. Models Involving the APP Pathway

APP is naturally cleaved into Aβ peptides by the sequential activities of the β- and γ-secretases [5]. PSEN1 and PSEN2 are subunits of γ-secretase, which participate in APP cleavage [66,67]. The classical AD amyloid hypothesis (with its periodic updates) proposes that there is an increase in Aβ production (particularly the longer forms of the peptide) or decrease of its clearance, with the increased concentration promoting Aβ aggregation [68]. The aggregates are found both intra- and extra-cellularly, and when outside the cell form Aβ plaques. Initial observations in AD patients and later in mouse models indicated the presence of “reactive” astrocytes stained with GFAP around Aβ plaques [28,69]. These early studies reported that most mouse models displayed some form of astrogliosis [69,70]—namely increased GFAP protein or *Gfap* gene levels in the cortex and hippocampus [26,71,72,73,74,75,76,77,78]. However, later studies showed that this finding varied in the context of the age, brain region, sex, and methods used to quantify GFAP or *Gfap* levels (e.g., Western blot or quantitative polymerase chain reaction, GFAP signal intensity of number of GFAP+ cells) in each model (Table 1 and Supplementary Table S1) [79,80,81].

The most studied AD mouse model, APPswe/PSEN1dE9, carries two transgenes, human *APP* with the Swedish mutation and human *PSEN1* lacking exon 9, both under the control of the mouse prion promoter (see Table 2). These mice develop plaque pathology and synaptic loss around 4 months of age [82,83,84], with cognitive impairment appearing by 6 months [82]. Astrogliosis is seen at 6 months, as assessed by increases in GFAP protein levels and in the GFAP+ cell number in the cortex of Tg compared to wild-type (WT) or younger mice [26,72,73,74,75,76,77,78,79,85]. Hippocampal samples of mice at the same age also showed elevated GFAP protein levels, a finding not noted in all studies [79]. These differences could be methodologic (e.g., measurements of GFAP) or biologic (e.g., sex related). In humans, females are more prone to developing AD [86]. In Tg2576 and 3xTg mouse models, males tend to develop plaque pathology later than female mice, while female 3xTg have more NFT pathology compared to males [87,88,89]. In APPswe/PS1dE9 mice, females have increased GFAP levels, reduced adult-born neuronal spines, and impaired spatial memory compared to males at 7–9 months of age [71]. Furthermore, GFAP+ astrocytes have increase branch length and number of branches in female APPswe/PS1dE9 mice versus WT, while such morphological changes are not observed in male transgenic mice at 7–9 months of age. Another example includes increased astrocytic gamma-aminobutyric acid (GABA) release by reactive astrocytes in the hippocampus and monoamine oxidase B (MAOB) activity, an enzyme which participates in GABA production, in 18-month-old female 5xFAD mice [90]. However, it should be noted that the above data (Aβ and NFT pathology, the astrocytic morphology and GFAP levels, and GABA levels) have not been validated in female versus male AD patients. Thus, while studies in mouse models suggest some potential explanations for the sex differences accounting for human AD prevalence, further investigation in human samples is necessary in order to validate the data. Additional complexity may also come from changes induced by housing conditions—as shown recently in amyloid mouse, rat, and *D. melanogaster* models of AD where microbiome changes caused different pathological and behavioral readouts (see [91] for review).

Apart from being used as a general marker for astrogliosis (i.e., measuring the number of cells expressing GFAP or signal intensity within a cell), GFAP has also helped characterize astrocyte morphology in the context of AD. The GFAP+ cell surface and volume were not significantly altered in the hippocampus of 5-month-old hAPP-J20 mice [97], which express human APP with the Swedish and Indiana mutations under the control of the human platelet-derived growth factor-β promoter. Nevertheless, the GFAP+ surface-to-volume ratio per cell was significantly reduced, indicating individual astrocyte GFAP morphology changes during progression in this model [97]. As GFAP can also stain astrocyte primary processes, the marker was used to measure the astrocyte branch length in the cornu ammonis 1 (CA1) and CA3 hippocampal areas of TgCRND8, which express human APP with the Swedish mutation and Indiana mutations, respectively, under the control of the hamster prion gene promoter. The GFAP+ branch length of the CA1 but not CA3 astrocytes at 3- and 6-month-old TgCRND8 mice was increased [103]. This is possibly due to the induction of reactive astrogliosis in the CA1, as assessed by the elevated GFAP signal intensity and number of GFAP+ cells. As astrocytes have distinct territorial domains that do not overlap extensively with neighboring astrocytes [104], it would be interesting to address how these domains are altered during AD and determine the functional outcomes of such morphological changes. Additionally, findings regarding astrocyte morphology in AD mouse models restricted to GFAP measurements, which is an intermediate filament protein, do not mark all of the astrocyte branching terminal processes, nor do they explain how the astrocyte function is changed. Therefore, GFP reporters or synaptic markers can be employed to analyze astrocyte morphology and complexity (i.e., Sholl analysis, coverage of synapses) [105]. Knowing that astrocytes undergo distinct morphological changes in disease contexts, detailed microscopy analysis could be pivotal in determining these alterations and how they are related to their altered function.

Thus, while we restrict the discussion largely to GFAP in this review, these data indicate that it is important to standardize experimental designs (e.g., age tested, environmental conditions) within a model in order to be able to make comparisons between studies.

Despite complications related to experimental standardization and GFAP, AD mouse models have been useful. The use of GFAP as an astrocyte marker extends to immunohistochemical colocalization with other markers to relate astrocytes with potentially AD-altered cellular functions. For instance, immunostaining of GFAP and Ki67 or BrdU, which are used to measure proliferation, did not cause colocalization in human AD or Aβ mouse models [78,123,124,125], suggesting significant proliferation of astrocytes does not occur in this context. Thus, it could be argued that the reported increased numbers of GFAP+ astrocytes in AD model mice and humans are most likely due to the induction of *Gfap* expression in GFAP- astrocytes. While GFAP, which is used as a broad marker of reactivity, has helped correlate changes in astrocytes with AD pathology in vivo (e.g., Aβ plaques), a disadvantage of using GFAP alone remains, namely that astrocytes maintain heterogeneous levels of GFAP across brain regions, development, and in multiple reactive sub-states [22,126,127]. What would be more useful for understanding astrocyte responses to AD pathologies would be unbiased transcriptomic or proteomic analysis of heterogeneous states and functional characterization of these subtypes where possible.

To gain better insight into the functional effects of Aβ and its aggregates on astrocytes in vivo, studies have addressed astrocyte functionality by measuring the electrophysiology, calcium transient parameters, gap junctional communication, glutamate uptake, expression and release of cytokines and growth factors, AQP4 localization, and Aβ uptake and clearance (Supplementary Table S1). Interestingly, there are conflicting results regarding Aβ uptake in vivo, despite the fact that studies have indicated that astrocytes can phagocytose Aβ in vitro and in situ and have identified mechanisms of action [128,129,130]. Additionally, Xiao et al. (2014) [128] drove exogenous expression of the transcription factor EB (*Tfeb*) in astrocytes (using the *Gfap* promoter) of 2-month-old APPswe/PSEN1dE9 mice. While TFEB induced lysosome biogenesis and enhanced uptake of Aβ42 by astrocytes in vitro, in vivo expression led to a decrease of PBS-soluble and -insoluble Aβ40 and Aβ42 fractions, probably via the same pathway. Further, immunohistochemical colocalization of GFAP and Aβ plaques and electron microscopy indicated engulfment of APP dystrophic neurites [95,99,100]. However, fluorescence-activated cell sorting (FACS) of MX04 (Methoxy-X04)-labelled plaques and tangles revealed Aβ within CD11B+/CD45+ microglia and not *gfaABC1D*-driven (a *Gfap*-derived synthetic promoter [131]) GFP+ hippocampal astrocytes at 9 months of age in APPswe/PSEN1dE9 mice [94]. Whether the difference is due to the methodology (MX04 stains Aβ plaques) or is a function of the studied model is not clear. Additional studies directly testing whether astrocytes phagocytose amyloid in vivo are necessary, and it should also be determined whether the mechanisms identified in vitro are also involved in Aβ uptake in vivo.

Aβ clearance across the BBB is well documented (for a review on Aβ clearance mechanisms see [124]), and astrocytes are implicated through their AQP4 water channels [132]. However, it has been shown that Aβ impairs AQP4 channel function, meaning that increased exposure to Aβ could feed back to impair its proper clearance in later stages of the disease [133]. In Tg-Arc/Swe transgenic mice (expressing human *APP* containing both the Arctic and Swedish mutation), an overall increase was shown in AQP4 protein levels in the cortex of 9-month-old mice compared to WT age-matched control animals [40,98]. The increase was preserved in older mice (up to 16 months old), as measured by AQP4 immunofluorescence intensity, although Western blotting showed no differences between the frontal cortex of 16-month-old Tg-Arc/Swe and WT mice [98]. The increase was accompanied by loss of AQP4 polarization within astrocytes close to Aβ plaques. Using electron microscopy, Yang et al. (2011) [40] characterized three stages of astrocytes surrounding Aβ plaques. GFAP+/AQP4- astrocytes were present at early plaque stages, followed by GFAP+/AQP4+, and lastly GFAP-/AQP4- astrocytes, which became more prominent in older mice. The functional relevance of these different populations was not addressed, but it seems that GFAP increases before AQP4. Future studies addressing whether the three astrocytic populations correlate with the stage of each plaque’s appearance could help in understanding how Aβ impacts astrocyte function. Perhaps the first two populations are different states of reactive astrocytes, while GFAP-/AQP4- astrocytes are atrophic. Studies in other models have measured AQP4 levels, the area covered, and AQP4’s association with vessels in order to gain further insight into Aβ’s effect on AQP4, but the results vary across models. The 12-month-old Tg2576 (transgenic expression of human *APP* containing the Swedish mutation) mice had identical numbers of AQP4+ astrocytic endfeet (stained with GFAP) in the frontal cortex and hippocampus compared to WT mice [101]. The same study found a significant reduction in both parameters in Tg-SwDI mice of the same age. The reduced association of astrocytes with vessels could be both indirect and direct. For instance, vascular amyloidosis can directly displace astrocyte endfeet from vessels [96]. Furthermore, the GFAP–vessel interaction is maintained in vessels without cerebrovascular amyloid angiopathy (CAA) in 16–22-month-old TgAPParc (human APP with the Arctic mutation) mice [102]. Since *Aqp4^−/−^* APP/PSEN1dE9 mice have increased Aβ accumulation [134], it is most likely that this accumulation impairs normal AQP4 levels and function, leading to enhanced Aβ pathology. It is not known if the findings in the different models also reflect possible differences among AD patients carrying distinct FAD mutations. Future studies are necessary to determine this and should determine the effects that different mutations have on specific astrocyte functions.

A substantial amount of progress has been made in examining the effect of Aβ on astrocyte function in vivo. However, there are still key knowledge gaps. For instance, we still do not know if the discrepancies regarding AQP4 findings between models reflect variability within the human population or if they are simply an artifact of our current rodent models. Most of these models express the transgenes (*APP*, *PSEN1*, *PSEN2*) above physiological levels and recapitulate some of AD’s pathological hallmarks, thus their translatability could be compromised. While some studies have attempted to validate results in both mice and humans—for example, GFAP/Ki67 colocalization analyses—others have not. Electrophysiological studies, for example, cannot be performed in post-mortem human brains, although it is possible to use human neuronal cultures, and in particular human iPSC-derived organoids [135,136,137]. Some of the recent methods and their discoveries regarding astrocyte function in AD will be briefly discussed later in Section 4. For an overview of astrocyte findings in APP and/or PSEN mouse models see Supplementary Table S1.

#### 2.1.2. Models Involving the *MAPT* Gene

Tau (encoded by *MAPT*) is a microtubule-associated protein expressed mostly in neurons. The degree and sites of Tau phosphorylation determine its ability to bind to microtubules and regulate their polymerization [138]. In AD, phospho-Tau increases and forms NFTs [139], causing disruption of axonal transport and cell death [139]. Mouse models carrying human *MAPT* mutations have increased phosphorylated Tau levels, many of which display neurofibrillary tangles, in contrast to APP and PSEN1 transgenic and knock-in mouse models, which do not (for an overview of these models see https://www.alzforum.org/research-models/alzheimers-disease.

Similar to amyloid models (see above), Tau phosphorylation and oligomer formation induces astrocyte reactivity in mice [140,141,142,143,144]. The age of onset for reactive astrogliosis, as measured by the signal intensity, area covered by GFAP, or number of GFAP+ astrocytes, varies across the Tau models and brain regions studied. These differences may manifest due to models being based on mutations, overexpression, and the spatial distribution of the transgene being expressed. For example, PS19 mice (expressing human Tau^P301S^ under the control of the mouse prion protein promoter) develop cortical astrogliosis as early as 3 months [140], while rTg4510 mice, which overexpress Tau^P301L^ mainly in forebrain neurons and show prominent neuronal loss and formation of NFTs [119,120,144,145], develop astrogliosis from 2.5 months of age [144]. In rTgTauEC mice, which overexpress Tau^P301L^ only in a subset of entorhinal neurons, increased number of GFAP+ cells in the entorhinal cortex is seen at 14 months of age [142]. In PS19 mice, astrogliosis in the dentate gyrus and the CA1 and CA3 regions of the hippocampus appears at 9 months of age (6 months later than in the cortex), as measured by GFAP-stained area [141]. Overall, the distribution and density of NFTs correlate with those of GFAP, suggesting a relationship between aberrant Tau and astrogliosis [117]. Interestingly, hyperphosphorylated Tau is present in the caudal cortex, entorhinal cortex, and CA3 from 3 months of age in PS19 mice, even though astrogliosis in CA3 develops later [141]. This could be due to selective vulnerability of different brain regions to AD pathology, which is further evidenced in rTgTauEC mice, where Tau pathology is seen in brain areas with direct connection to the entorhinal cortex (the hippocampal and para-hippocampal areas, the amygdala, and the perirhinal cortex) in the absence of measurable astrogliosis (although it is present in the amygdala) [142]. Thus, it seems that the presence of aberrant Tau forms does not always cause immediate astrogliosis. 

The presence of more well-defined astrogliosis in Tau models has been shown using unbiased RNA sequencing of hippocampal astrocytes [33]. Here, PS19 astrocytes upregulated genes associated with a pro-inflammatory A1-specific set of genes, as well as genes associated with several reactive states and genes involved in the classical complement pathway (*C1q*, *C4b*, *C3*, and *Serping1*), which could contribute to neurodegeneration. Silencing C3 (a complement component involved in inflammation and cell lysis [146]) using global knock-out (*C3^−/−^*) prevented Tau-induced neuronal death in PS19 mice [33]. It was also reported that in *C3^−^*^/*−*^ x PS2APP (human APP with the Swedish mutation and PSEN2^N141I^) mice, neuronal synaptic loss in proximity to Aβ plaques is reduced. Additionally, C3 receptor, C3aR, mRNA, and protein levels are increased in PS19 mice [147]. *C3ar^−^*^/*−*^ partly reduces the Tau pathology and GFAP+ area in 9-month-old PS19 mice, among others. Mechanistically, the C3 receptor, C3aR, induces STAT3 phosphorylation [147]. When the transcription factor STAT3 is phosphorylated, it translocates to the nucleus to induce transcriptional changes in some subsets of reactive astrocytes [22,148]. Nevertheless, the *C3^−^*^/*−*^ rescue in PS2APP and PS19 mice may not have been astrocyte-specific, since microglia, which are also implicated in neuroinflammation and neuronal death, can upregulate *C3*. Microglia and astrocytes interact in the brain, and activation of microglia can induce astrocyte reactivity, which is predicted to drive neuron death [30,149]. Thus, the presence of Aβ or Tau aggregates is likely to trigger both cell types to become reactive. The exact mechanisms of Aβ- and Tau-induced astrocyte and microglia activation are not yet known, but these results further suggest that targeting the inflammatory cascade could be a valid strategy for drug development in AD.

Tau hyperphosphorylation is reported in brain regions directly connected with the entorhinal cortex in rTgTauEC mice [142]. This is because Tau aggregates, which are largely composed of the phosphorylated protein, can be transmitted from one neuron to another [150,151,152] through possible synaptic vesicle release across the synaptic cleft [153]. Astrocyte endfeet are in close association with these synaptic clefts (forming the “tripartite” synapses [17,154]), suggesting they may also play a role in Tau seeding. Tau uptake by astrocytes is evident in in vitro cultures [155], and in AD patients it appears that some astrocytes contain NFTs [156]. To test whether aberrant neuronal Tau can be transmitted to astrocytes, cortical and hippocampal brain sections from Tau^R406W^ transgenic mice (which express human Tau in excitatory neurons under the control of the *CamkII* promoter) were used [157]. Using this mouse model, astrocytes did not stain with antibodies specific for human Tau. However, astrocytes did contain mouse Tau. Similarly in Tau^P301L^ mice, expressing human Tau in neurons under the control of the *Thy1* or mouse prion protein promoters, saw GFAP+ astrocytes contain or associate with oligomeric and filamentous human Tau [158,159], implying that some astrocytes indeed show Tau pathology. However, Tau aggregates from human AD patients do not display Tau inclusions in glial cells [158], thus additional experiments are necessary to identify the extent to which Tau uptake occurs in AD, which Tau forms are taken up by astrocytes, and by which mechanism. Do different *MAPT* mutations influence astrocyte uptake of Tau? What is the effect of aberrant Tau formation in astrocytes? Does astrocyte-expressed Tau, albeit at low levels, contribute to AD pathology? These are some of the questions that should be answered to gain a better understanding of Tau-induced astrocyte pathology.

Similar to models that only contain *APP*/*PSEN1* mutations, mice bearing only a mutant *MAPT* gene may not fully recapitulate astrocyte functional deficits. Tau models provide a great tool to understand how abnormal Tau levels or forms impair normal astrocyte function, but caution should be taken when translating findings to human AD, where there is extensive Aβ pathology and Tau phosphorylation appears to be a downstream event. To better model AD pathology, models with mutations in *APP*, *PSENs*, and *MAPT* genes have been developed. One such model, the triple transgenic model (3xTg, knock-in of human PSEN1^M146V^, and transgenic expression of human APP with the Swedish mutation and human MAPT^P301L^), has been extensively used to study astrocyte pathology in AD (see Supplementary Table S2). Astrocytes around Aβ plaques become reactive (increased GFAP levels [25,160,161,162,163,164] and cytokine production [164,165]). Nevertheless, such findings only summarize the astrocytic response. Bronzuoli et al. (2019) [160] compared the common astrocyte markers, GFAP, S100 calcium-binding protein B (S100β), connexin 43 (CX43), and AQP4, in the hippocampi of transgenic and WT mice at 6 months (plaque appearance) and at 12 months of age (NFT appearance). Interestingly, reduced GFAP, S100β, CX43, and increased AQP4 protein levels occurred in 12-month-old compared with 6-month-old mice, irrespective of the brain region. This is one of the many studies showing that glial response in AD varies across brain regions and with age, with the authors opining that there is a complex relation between aging and AD. To gain a better understanding of astrocyte response at single-cell resolution in FAD and the role of aging, more molecularly detailed studies are required (see transcriptomic studies below).

### 2.2. Sporadic or Late-Onset Alzheimer’s Disease Mouse Models

Most human AD is of sporadic or late onset on a range of genetically conditioned backgrounds, perhaps involving presently unknown environmental factors. The heritability estimate for LOAD is 60–80% [8], indicating that genetic predisposition is a major factor for developing this multifactorial disease. As noted, *APOE* is the major genetic risk factor for developing LOAD [60], with other inflammation-associated genes identified in GWAS databases containing DNA from hundreds of thousands of LOAD subjects. Some of these, notably *Apoe* and *Trem2*, have been used to examine the influence on normal brain function. There are other genetically induced LOAD mouse models available (https://www.model-ad.org/strain-table/), but due to space limitations they are not all listed here.

#### 2.2.1. Apolipoprotein-E Models in Alzheimer’s Disease

APOE is a lipid-associated protein that transports lipids, mostly high-density lipoprotein, between cells and organs [166]. In the CNS, *APOE* is mainly expressed by astrocytes [167] and is necessary for transport of cholesterol to neurons. The different human *APOE* alleles (ε2, ε3, and ε4) differentially predispose individuals to developing AD [60] (with ε2 being protective, while ε4 increases susceptibility). ApoE mouse models have been made in one of three ways: (1) by knocking out the endogenous mouse *Apoe* gene to create null mice, (2) by targeted replacement of the mouse *Apoe* gene with the human *APOE2*, *3*, or *4* allele, or (3) by overexpressing human *APOE2*, *3*, or *4* under a promoter such as *GFAP*. The substitution of human for mouse is not sufficient to induce astrogliosis, as measured by the number GFAP+ cells in the hippocampus of 4–6-month-old *APOE-*KI mice compared to *Apoe^−^*^/*−*^ mice [168]. The levels of IL-1β, IL-6, and TNF-α pro-inflammatory cytokines are also similar among the three strains. This may be due to age, as these mice may have been too young to observe any genotype-related differences, or due to inherent species differences between humans and rodents. For a better understanding of how human APOε4 influences different cell types in the CNS, Tesseur et al. (2000) [58] developed mouse models where *APOE4* transgene overexpression was controlled by the mouse *Thy1*, or by human platelet-derived growth factor (*Pdgf*), *Gfap*, or phosphoglycerate kinase (*Pgk*) promoters. Neuron-specific transgene overexpression of *APOE4* (mouse *Thy1* and human *Pdgf* promoters) led to an observed increase in the number of GFAP+ astrocytes compared to mice overexpressing human *GFAP-* and *PGK-*driven *APOE4* and to WT mice (8 and 18 months of age). However, the findings in the different mouse lines could have been caused by differential expression levels of *APOE4* between the models. For instance, human *PGK*-*APOE4* mice have lower APOE protein levels compared to the other transgenic mice, which could explain the absence of astrogliosis. Additionally, the onset of *APOE4* transcription varies by promoter, and since cholesterol is involved in brain system development [169], the results could reflect developmental rather than adult functional effects. However, *PDGF* is active from embryonic day 9.5 (human *PDGF* promoter sequence was used in the study) and *Thy1* from postnatal day 15 (Table 1 in [58]), suggesting that the observed increase in the number of GFAP+ astrocytes reflects prenatal effects—perhaps an increase in astrocyte proliferation or differentiation. Conclusively, most of the findings in ApoE mouse models point towards a less robust impairment of astrocyte function compared to FAD mouse models. This is in accordance with the consideration of APOE as an AD risk factor. APOE seems to sensitize the brain to developing AD, but there are insufficient data on whether replacement of mouse with human *APOE* could lead to AD-like pathology in the carriers.

To better address how the *APOE* genotype influences astrocytes during AD development in vivo, ApoE models have been combined with FAD models. The cortical and hippocampal GFAP protein levels, number of GFAP+ astrocytes, and GFAP+ astrocytes per Aβ plaque are reduced in *Apoe^−^*^/*−*^ × APPswe/PSEN1dE9 and *Gfap*-driven *Apoe^−^*^/*−*^ × APPswe/PSEN1dE9 compared to APPswe/PSEN1dE9 mice at 12 months of age [170]. *Apoe^−^*^/*−*^ reduces Aβ plaque deposits, and consequently astrogliosis (number of GFAP+ astrocytes) of PDAPP mice overexpressing the *APP* transgene with the Indiana *APP* mutation [171]. Thus, the absence of *Apoe* ameliorates AD-induced astrogliosis. In contrast, human APOE-KI (ε2, ε3 or ε4) in 5xFAD mice shows prominent astrogliosis in the subiculum and deep cortical layers at 6 months [172], although the effect of each allele on astrocyte activation was not measured. To compare how the *APOE* genotype influences Aβ-induced astrocyte pathology, Dorey et al. (2017) [173] found that protein levels of several cytokines (interferon-gamma (IFNγ), monocyte chemoattractant protein-1 (MCP-1), macrophage inflammatory protein (MIP-1a), Skp/Cullin/F-box containing complex (SCF), Rantes, beta nerve growth factor (b-NGF)) were increased in *GFAP*-*APOE4* (transgenic overexpression) × APPswe/PSEN1dE9 versus *GFAP*-*APOE2* (transgenic overexpression) × APPswe/PSEN1dE9 mice at 6 months of age. These results provide additional evidence that *APOE4* sensitizes the brain to developing AD. To gain more functional insight into pathways involved in APOE-induced astrocyte activation in a FAD background, Zheng et al. (2017) [170] measured the protein and mRNA levels of STAT3/*STAT3*, phospho-STAT3, SMAD2/*SMAD2*, and phospho-SMAD2 in *Apoe*^−/−^ and *GFAP*-driven *Apoe*^−/−^ × APPswe/PSEN1dE9 compared to APPswe/PSEN1dE9 mice. STAT3 [22,148] and the TGF-β/SMAD2 pathway [22,174] are involved in inducing reactive astrogliosis. The ratio of both pSTAT3/STAT3 and pSMAD2/SMAD2 and the TGF-β protein levels are reduced in *Apoe*^−/−^ × APPswe/PSEN1dE9 and *Gfap*-*Apoe*^−/−^ × APPswe/PSEN1dE9 compared to APPswe/PSEN1dE9 mice at 12 months of age [170]. Nevertheless, these measurements in both *Apoe*^−/−^ × APPswe/PSEN1dE9 and *Gfap*-*Apoe*^−/−^ × APPswe/PSEN1dE9 models are higher than in WT, suggesting only a partial rescue by knocking out *Apoe*. The effects of *APOE* genotype on astrocytes could be related to APOE-driven Aβ uptake from neurons. Perivascular astrocytes, which can clear Aβ from the brain into the perivascular space, show colocalization with human APOE and Aβ in APP^V717I^/PSEN1^A246E^ mice when the *APOE*, *APP*, and *PSEN1* transgenes are overexpressed in neurons by the *Thy1* promoter [175]. The astrocytic response to aberrant Tau can also be influenced by the *APOE* genotype. Similar to APP models, human *APOE4*-KI astrocytes become reactive by upregulating “A1”-reactive genes in the cortex of 9-month-old human *APOE4*-KI × Tau^P301S^ versus *Apοe^−^*^/*−*^ × Tau^P301S^, further suggesting that APOE worsens AD pathology [176]. The study found a correlation between an elevated hippocampal or piriform GFAP+ area and decreased hippocampal or piriform volume, respectively. It should be noted, however, that this study investigated expression levels of a small subset of genes indicative of only one subtype of reactive astrocytes and that there are likely heterogeneous populations of astrocytes present at the same time, as reported recently in other mouse models of AD [31,36]. Thus, the genetic APOE state (i.e., ε2, 3, or 4) seems to be related to the degree of reactive astrogliosis via the classical astrocyte activation pathways in APP mouse models, although additional in vivo evidence regarding the precise astrocytic molecular mechanisms engaged by Aβ or Tau and the downstream effects is lacking. Once such mechanisms are identified, their presence should be explored in human AD patients to determine if they are a potential therapeutic target. 

#### 2.2.2. TREM2 Models in Alzheimer’s Disease

Triggering receptor expressed on myeloid cells-2 (TREM2) is a genetic risk factor for human AD and is expressed by microglia and infiltrating myeloid cells in the CNS [177]. *TREM2^−^*^/*−*^ [178] x PS19 9-month-old mice have reduced *Gfap* expression and a reduced GFAP-stained area, and the correlation between GFAP- and the IBA1-stained area in the hippocampus is lost compared to PS19 mice, indicating that microglia have an important role in Tau-induced astrogliosis [179]. The expression of *Il1a*, *Il1b*, *Tnf*, *C1q*, and *Apoe* in the cortex was also reduced, suggesting that TREM2 aggravates AD pathology. For a more thorough understanding of the effect of *Trem2*^−/−^, Zhou et al. (2020) [36] performed single nucleus RNA sequencing in 7-month-old *Trem2*^−/−^ × 5xFAD, *Trem2*^−/−^, 5xFAD (expressing *APP* with the Swedish, Florida, and London mutations and *PSEN1*^M146L,L286V^ under the control of the mouse *Thy1* promoter), and WT mice. The study identified genes that were TREM2-dependent and TREM2-independent of Aβ pathology. Unsurprisingly, given the expression of *Trem2* almost exclusively by microglia, the most robust and significant changes were seen in microglia rather than in neurons or astrocytes (although there were considerable transcriptional changes in oligodendrocytes). Additionally, no robust transcriptional changes were seen in human *TREM2^R62H^* (a mutation that increases risk for developing AD) astrocytes from AD patients. A more evident effect on astrocytes was observed in human AD samples expressing WT (non-mutated) TREM2, where astrocytes downregulated genes involved in lipid and oxidative metabolism compared to control samples. Given the transcriptomic differences between human AD with WT TREM2 and *TREM2^R62H^*, it would be interesting to gain more functional insight on how the background of *TREM2* influences astrocyte reactivity in AD. The above paradigms highlight the complexity of AD. TREM2 is a risk factor for developing AD, and microglial pathogenesis and pathology are not entirely dependent on TREM2. The findings between human AD and mouse models attempting to recapitulate AD show some dissimilarities. Additional studies in the future should address how TREM2-dependent and -independent microglia mechanisms influence astrocyte activity in AD and should characterize which FAD models are the most relevant for modeling TREM2-dependent responses in mice.

Both *APOE* and *TREM2* are risk factors for developing LOAD [60,61,62,63]. Owing to a focus on genes implicated in FAD (*APP*, *PSENs*, *MAPT*), mouse models with human *APOE*-targeted replacement and *Trem2*^−/−^ have been less well characterized than FAD models (currently less than 2000 studies in PubMed for *APOE* and *TREM2* compared to over 15,000 studies for APP or Tau mouse models—see Supplementary Information). It seems that a FAD background is necessary to characterize the susceptibility or resistance to AD of LOAD models. Perhaps mutations in multiple risk factor genes are necessary. Even if this is the case, environmental factors that could increase or reduce the onset of AD are not present in mouse studies. For an overview of how AD factors impair normal astrocyte function, see Figure 1. The development of new models will require their proper characterization, and as astrocytes have only recently gained attention in AD (and in general), there are limited data regarding their behavior under normal and pathological conditions in mice and humans. In the next section, the main limitations of current mouse models will be discussed to provide a better understanding of how to proceed in the future. For an overview of astrocyte findings in LOAD (APOE, TREM2) mouse models, see Supplementary Table S3.

## 3. Limitations of Studying Astrocytes in Mouse Models

### 3.1. Limitations Restricted to Astrocytes

One of the major problems of mouse models is translatability, which is partly explained by differences between human and mouse astrocytes. Recent studies using morphological or transcriptomic comparisons are beginning to characterize the differences [180,181]. Such differences could affect AD progression and pathology in each species. For instance, human protoplasmic astrocytes are morphologically more complex and have a higher degree of physical overlap among cells than those of mice [181]. It is possible that the increased overlap could increase Tau seeding or accelerate progression of pathology. Furthermore, in humans there is increased astrocyte complexity and increased coverage of multiple neuronal processes per astrocyte (i.e., GFAP+ domain diameter of 142.6 ± 5.8 μm in humans versus 56.0 ± 2.0 μm in mice for protoplasmic astrocytes [181,182]). Aβ pathology leads to astrocytic atrophy away from plaques [71,73,77,80,183], meaning fewer neuronal processes are covered. A testable hypothesis is that astrocytic atrophy is part of a defense designed to minimize seeding of Aβ or Tau aggregates. Despite the presence of atrophic astrocytes prior to disease onset that are hypothesized to lose their function [9], there is no evident impairment in cognition during early AD stages in humans. Perhaps individual astrocytes respond differently to Aβ, Tau, and inflammation, and these subpopulations might be different between humans and mice. An example indicating that human and mouse astrocytes respond differently to AD pathology is presented in a single-nucleus RNA sequencing study by Zhou et al. (2020) [36]. A population of astrocytes with high expression of genes involved in lipid and oxidative metabolism under normal conditions was lost in human AD cases, but remained intact in 5xFAD mice. Whether this is due to differences in mouse and human astrocyte responses to pathology or is an artifact of the model is unknown. Additionally, a comparison of mouse and human astrocyte-enriched genes revealed that 52% of mouse and 30% of human genes were identically enriched in human and mouse astrocytes, respectively [180]. Whether there are functional differences between human and mouse astrocytes in adults is yet to be determined—although there are examples of similarities [137,180].

Species differences between astrocytes from humans and mice may limit the translatability of some findings. The best example is the expression of Toll-like receptors (TLRs). TLRs are activated by pathogens or danger-associated molecular patterns (DAMPs), including Aβ [44], triggering an inflammatory response [184,185]. TLRs are expressed in all cell types in the CNS, although different family members may be differentially expressed in different cells [186]. As inflammation plays an important role in AD, so do TLRs (for a review see [186]). Many studies on TLRs and inflammation have been done in mice, however the members and level of expression of each receptor varies between mouse and human CNS cells. For instance, human astrocytes express *Tlr1-6* and *Tlr9*, whereas mouse astrocytes express *Tlrs* at low levels or not at all [65,180,186]. Additionally, the recognition pattern for bacteria between TLR9 in mice and humans differs [187]. Thus, the genetic differences of mice compared to humans can explain why there is a lack of translatability. Currently, other models based on a wider number of mouse backgrounds (https://www.model-ad.org/strain-table/) and human-induced pluripotent stem cell (hIPSC) culture models ([188,189,190] among others) are being used and developed to better understand human AD pathology.

### 3.2. Other Limitations of Studying AD in Mice

Aging is the major risk factor for contracting AD [191]. Mouse and human transcriptomic studies have characterized age-related changes in the brain [149,192,193]. Furthermore, each AD mouse model develops the pathology at a different age (for an overview visit https://www.alzforum.org/research-models/alzheimers-disease). Thus, the interaction with aging contributes differently to each transgenic or knock-in mouse line, adding another level of complexity. The mouse genome and aging are not sufficient for AD to naturally occur in mice. Directly comparing aging between different organisms may also be complicated due to species-specific differences. These original species comparisons of astrocyte transcriptomes were done using non-comparable brain regions, so they may represent regional differences as well as species differences. Additional studies report some similarities in transcriptomic changes in mouse and human CNS cells with age [149,192,193]. Perhaps comparing such studies can help pinpoint differences between mouse and human aging and identify which mouse models most closely resemble the time point of AD onset in humans.

Apart from these core limitations, there are others that deserve attention. More women suffer from AD than men, and AD mouse models show sex-related differences in plaque load and NFT pathology [86,87]. The use of both sexes interchangeably (or in combination) in the past has restricted our understanding of AD vulnerability between sexes. Furthermore, the number of males to females in each experimental group was not always mentioned, rendering comparisons among or between studies difficult.

Housing imposes another variable that must be controlled for when using mouse models. To reduce inter-individual variability, mice are bred in a controlled environment. These controlled environments remove exposure to environmental factors—these are known to alter the gut microbiome, which can robustly change the pathogenesis of some APP transgenic mouse models [194,195,196]. Similarly, gut bacterial flora can interact with the inflammatory system, which consequently led to astrocyte and microglia changes in an experimental autoimmune encephalomyelitis model [197]. Since gut microbiota seem to play a role in AD development [91,198], it is possible that environmental factors affect the results and could account for discrepancies between laboratories. The recently advanced notion that Aβ behaves similarly to a microbial defense mechanism makes these considerations even more relevant [199].

Importantly, the mice themselves are inbred, meaning that genotype differences between mice are kept to a minimum [200]. While this approach ensures findings and treatment effects can be reproduced between laboratories, the human population consists of high genetic variability. Any treatment developed in the context of a single mouse model might only be relevant for a specific population of humans. On the other hand, effects seen in mice might not be observable in humans due to the individuals selected and the genetic differences between humans and mice. Similarly, mouse models with different APP mutations show different findings (e.g., in AQP4 [40,98,101,201]). These differences might be a model artifact or could be mutation-related and represent the different vulnerability of individuals to developing AD (affected by components or pathways). 

Combined, age, sex, and microbiome present additional complications that have not been fully recognized with respect to astrocyte responses in diseases such as AD. These variables are now being acknowledged with respect to our full understanding of the heterogeneous and context-dependent responses of all glial and immune cells to disease, infection, and injury.

## 4. Recent Studies and Future Perspectives for Studying Astrocytes in AD

### 4.1. Transcriptomic Studies

As mentioned above, human and mouse astrocytes have similarities, but also important transcriptomic differences. The heterogeneity is also present within a single species. Hippocampal astrocytes differ from cortical astrocytes in their morphology, transcriptome, proteome, electrophysiology, and Ca^2+^ signaling [126]. Morphological differences between astrocytes in different cortical layers in the mouse suggest subregional heterogeneity [105], which has been confirmed transcriptomically in both mice and humans [127]. High-throughput in situ hybridization shows both layer-dependent and -independent heterogeneity. Additionally, not only do astrocyte transcriptomes differ spatially (between and within different brain regions), but also temporally (throughout aging) [149,193]. Studies that are designed to identify the extent to which various astrocyte sub-states are predetermined or locally plastic, as determined by their anatomic or functional niche, and how aging affects each sub-state would shed even more light on our understanding of selective vulnerability in AD. Such information could also be used to identify mechanisms of resistance that might be enhanced therapeutically to arrest or reverse the pathology.

Whereas these studies focus on astrocytes under normal conditions, it should be noted that transcriptomics are also used to decipher astrocyte response in disease contexts, including for AD. Habib et al. (2020) [31] identified a population of astrocytes with a unique transcriptomic signature (termed disease-associated astrocytes—DAAs) appearing at 4 months of age in 5xFAD mice. This population was found in WT animals from 13 months of age, but even at 20 months of age the number of DAAs was minimal compared to 7-month-old 5xFAD mice. DAAs express high levels of *Gfap* but have different enriched pathways compared to high-expressing *Gfap* astrocytes present in normal conditions. The lysosomal, inflammatory response, and complement pathways are few of the DAA-only enriched pathways in 5xFAD mice. Furthermore, the expression profile of DAAs partially matches the pan-reactive and A1-reactive profile of astrocytes in the Zamanian et al. (2012) [29] and Liddelow et al. (2017) [30]. It would be interesting to see whether DAAs are associated with plaque pathology or if they are present in human patients. Chen et al. (2020) [32] conducted spatial transcriptomic analysis and in situ sequencing in APP^NL-G-F^ mice, comparing not only the genotype (APP^NL-G-F^ or WT), but also the effect of Aβ plaques on gene expression. The most differentially expressed genes in both categories were grouped and termed plague-induced genes (PIGs). PIGs are mostly enriched in microglia and to a lesser degree in astrocytes. Interestingly, as the disease progresses and Aβ accumulates, the network connectivity of microglial and astrocytic genes increases. For example, astrocytic cathepsin D, a lysosomal protease whose gene expression is deregulated in AD [202,203,204], forms strong connections with microglial complement pathway components *C1qa* and *C1qb*, which are involved in astrocyte reactivity induction [30]. Astrocytic *ApoE* also forms strong connections with microglial *C1qb* [32]. ApoE binding to C1q near Aβ plaques leads to inhibition of the complement cascade and correlates with cognitive decline [205]. Additional functional studies are necessary to identify how AD-related genes, which were characterized in various transcriptomic studies, interact during disease progression and whether they could be targeted for treatment.

In different astrocyte transcriptomic studies in AD mouse models, researchers have compared the astrocytic expression to the A1- or A2-reactive astrocyte profile [31,32,33]. However, only a partial similarity was observed. Additionally, the signatures of A1- and A2-reactive astrocytes have not been validated in human AD brains or genetically induced AD mouse models. Das et al. (2017) [206] performed a meta-analysis of mouse astrocyte transcriptomes in acute injury and neurodegenerative disease mouse models, including the APPswe/PSEN1dE9, PS2APP, and PS19 AD mouse models. They found that whether an astrocyte will acquire an A1 or A2 transcriptomic phenotype is not specific to the disease’s nature (acute injury versus neurodegeneration). Rather, genes from each category are upregulated in each disease. This further suggests summarizing the astrocyte response into A1- or A2-reactive could lead to data misinterpretation. This does not mean there are not distinct astrocyte transcriptome signatures between different diseases. For instance, mouse models of acute CNS injury display different transcriptome profiles to mouse models of neurodegeneration. It is highly likely that such a heterogeneity is present between different models of acute injury or neurodegeneration (i.e., Alzheimer’s disease, Parkinson’s disease, amyotrophic lateral sclerosis), yet further studies are necessary to validate this. Conclusively, astrocyte transcriptomic analyses in AD mouse models are a powerful tool for pinpointing genes and pathways involved in the disease progression, but care should be taken when extrapolating the results to humans (see [206]). The above studies reported astrocyte heterogeneity within a few brain regions, while Wang et al. (2016) [207] performed a transcriptomic network analysis of 19 human brain regions, comparing AD and control patients. The study highlights differentially expressed genes per brain region with AD pathological traits—including cognitive deficits, Aβ plaques, and NFT pathology. Of these regions, 17 had modules enriched for inflammatory responses—a common response of astrocytes in many neurodegenerative diseases and infections ([30,149,176,208,209], among others). If inflammation is commonly induced in many brain regions, why do some cortical regions and the hippocampus develop AD pathology first? Is it due to the differential expression of inflammatory genes between brain regions or the contribution of other pathways such as lipid homeostasis (see [193]) (or both) that explain this vulnerability? To what extent does brain area connectivity contribute to spatial vulnerability? The heterogeneity in astrocyte responses—across brain regions, throughout development, and with respect to the initiating stimuli—may explain the selective vulnerability of different brain regions to developing diseases such as AD. Some possibilities are supported with more evidence than others, but in the end integration of (new) transcriptomic studies with other already published datasets can provide a basis for forming new hypotheses to study how cellular—in our case astrocytic—homeostasis is impaired in AD. These studies will provide a better understanding of not only disease pathology, but also whether there is a common mechanism in AD that could be targeted for treatment.

### 4.2. The Rise of Proteomics

While transcriptomics have become relatively more accessible, their lowered costs still remain beyond the means of many groups. Proteomic studies might capture additional pathways that are activated by post-translational mechanisms and do not require changes in transcription. Using mass spectrometry (MS)-based proteomics in human samples of the dorso-lateral prefrontal cortex with or without AD, Johnson et al. (2020) [210] generated an AD brain coexpression network to pinpoint pathways and cell-type-specific changes. The study correlated protein levels with Aβ plaques, NFTs, and cognitive functions to identify relationships independent of Aβ and NFTs. One of the most significantly altered biological functions involved glucose and carbohydrate metabolism. This module consisted of astrocytes and microglia, which upregulated several anti-inflammatory genes (i.e., *Fabp5 Spp1*, *Lmnb1*), although a few pro-inflammatory genes were also identified (i.e., *Tspo*, *Iqgap1*, *Nampt*). Microglial proteins highly present in AD samples were involved in anti-inflammatory response, while astrocyte markers included both A1- and A2-reactive gene products. As in AD, normal aging brains showed an increase in the levels of proteins involved in glucose and carbohydrate metabolism. However, other modules were differentially affected, implying a complex relationship between aging and AD at the protein level. The study is limited to the dorso-lateral prefrontal cortex. In the future, more brain areas should be analyzed using proteomics to gain a better understanding of region vulnerability in AD, which could also help target potential pathways for treatment. 

Proteomics has also been used to study the cerebrospinal fluid (CSF) for biomarkers with possible clinical or pathogenetic significance [210,211]. Glucose and carbohydrate metabolism pathway-related proteins were enriched in the CSF of AD patients, with increasing levels observed in asymptomatic AD patients for some of these proteins [210]. Future studies should test whether the pathway of glucose and carbohydrate metabolism is similarly affected in other brain regions. Additionally, they should also validate the extent to which the findings reproduce the results of transcriptomic studies. Currently, single-cell proteomics studies have addressed human microglia heterogeneity [212], and perhaps similar data regarding CNS cells, including astrocytes, in AD will be helpful towards our understanding of this disease. 

Transcriptomics and proteomics provide great tools that can be used to identify novel pathways and biological processes in AD, but the findings should be validated by functional studies. In vitro cultures and in vivo mouse models are still necessary to understand astrocyte function in a simple cellular model (in vitro) and in a model with the complexity of a living organism (mice and other animal models [213]).

### 4.3. Novel Mouse Models

As most AD is sporadic, and many recent studies have identified genetic loci implicated in human LOAD, relevant mouse models for LOAD using these genes are being developed in the context of the Model Organism Development and Evaluation for Late-Onset Alzheimer’s Disease (MODEL-AD) consortium (https://www.model-ad.org/). This project is tasked with creating novel in vivo mouse models for LOAD [46]. It is hoped that the use of multiple mouse strains will provide genetic diversity that is more similar to that in human patients [213]. For example, it is known that C57BL/6J appears to be resistant to AD and similar neuropathologies, however the mechanism is unknown. The long-term goal of MODEL-AD is to characterize such models, defining features that correspond to stages of the human disease in order to discover biomarkers related to pathogenesis that can be used in drug development and clinical testing. MODEL-AD attempts to overcome the problems intrinsic to the prior models, which are based on the role of Aβ in pathogenesis. It must be noted that since LOAD and FAD have been defined by the presence of plaques and tangles, it may be difficult to produce those pathologies in the absence of human AD-associated transgenes. Similarly, models based on the administration of streptozotocin intracerebrally, or of LPS systemically or intracerebrally, while reproducing many of the inflammatory features of AD and other neurodegenerative diseases, have only minimal increases in Aβ (plaques) or Tau aggregation (tangles). This makes them difficult to interpret in the context of human LOAD, which shows both.

Currently, there are no data describing astrocyte function in MODEL-AD mouse models. Presumably, after standardization and extensive characterization of mouse models, such studies will shed light on the role of astrocytes in LOAD. Since multiple genetic loci are implicated in LOAD, different models should provide evidence of the different aspects of astrocytes in AD pathophysiology. Additionally, mixing these models could further contribute to how different genes and cellular pathways might induce abnormal astrocyte function in LOAD. While no mouse model will be able to recapitulate all aspects of astrocyte function (or dysfunction) associated with AD, proper characterization of the transcriptomic, proteomic, and functional changes present in current and future models and comparisons with human data are required. Animal models will remain necessary to understand AD pathology and astrocyte function, particularly in vivo [214]. 

### 4.4. Chimeric Mice

**Figure 2:**
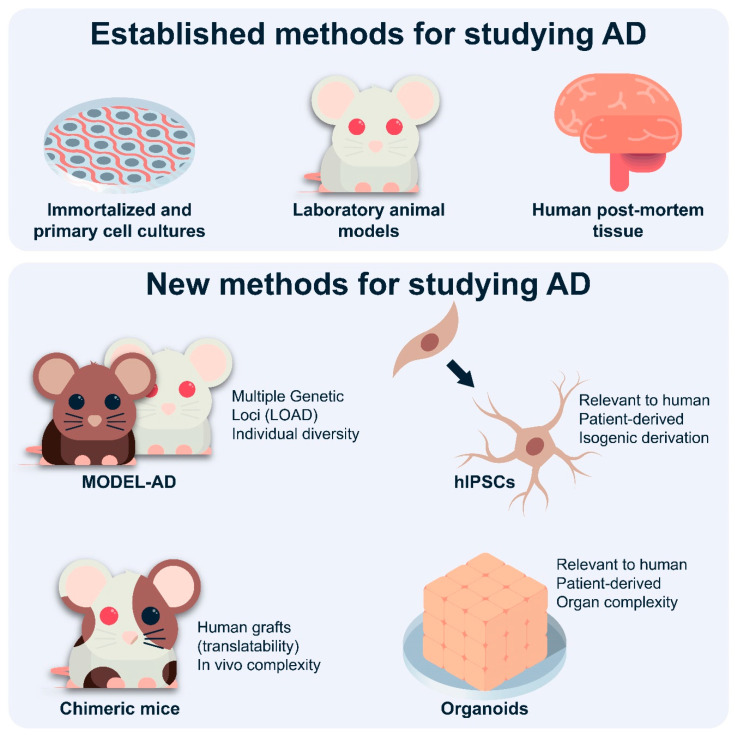
Overview of established and novel methodologies and models used to study Alzheimer’s disease. Top: various in vitro and in vivo models have been generated over the past years and are being used along with human tissue to study AD. Bottom: four new methodologies and models that have been developed in the past few years to improve the translatability and relevance of findings for AD. The MODEL-AD consortium will increase the availability of mouse models carrying different mutations and will include wild mouse strains that represent the genetic diversity present in human patients. Human-induced pluripotent stem cells that can be differentiated to a specific cell type (i.e., astrocytes) or organoids are in vitro models with a human genetic background and can be specific for each patient. Chimeric mice where hIPSCs or human differentiated cells can be engrafted in an in vivo model can combine the advantages of a living organism—in this case a mouse—and human in vitro models. Note that for the new methodologies, the main advantages for AD are mentioned, but there are also major drawbacks. For additional information on the models for studying brain disease, the reader can refer to specific reviews (MODEL-AD (https://www.model-ad.org/), hIPSCs [219,220,221], organoids [136], chimeric mice [215]). Abbreviations: AD = Alzheimer’s disease; hIPCS = human-induced pluripotent stem cells; LOAD = late-onset Alzheimer’s disease; MODEL-AD = Model Organism Development and Evaluation for Late-Onset Alzheimer’s Disease.

Mice are genetically different to humans, which often limits the translatability of AD mouse models. To partially overcome this, chimeric mice, whereby human glial progenitor cells (hGPCs) are engrafted in the mouse brain, can be used (Figure 2). Indeed, the hGPCs engrafted in the neonatal mouse brain can expand and differentiate into astrocytes and oligodendrocytes [182]. In *Shiverer* mice, which display hypomyelination, human glia replaced the host population within a year [182]. These mice ought to help us understand how human glia contribute to neurodegenerative disorders. The chimeric glia mouse brain usefulness and limitations in studying the glial role in human pathology have already been reviewed [215]. So far, such chimeric mice were used to identify the glial role in Huntington’s disease [216] and childhood schizophrenia [217]. Other than understanding their pathogenic role in neurodegeneration, chimeric mice could be used to understand how human astrocytes contribute to increased cognition. Han et al. (2013) [218] engrafted hGPCs into neonatal mice, which showed improved synaptic plasticity and learning. The differences between mouse and human astrocytes in the mouse brain could perhaps help identify potential reasons why mice do not develop AD. Other potential uses of chimeric mice include engraftment of hGPCs carrying FAD mutations. The mutation could occur naturally and represent the natural variability of human AD or could be induced by mutagenesis to ensure an isogenic background between mutated and control lines. For LOAD, APOε4 or ε3 hGPCs could be used. Of course, other genes implicated as risk factors for LOAD can be studied. Since hGPCs require human fetuses, it would be helpful to develop a protocol for grafting hIPSC-derived hGPCs in mouse brains. The field of study for chimeric mice is relatively new and is believed to provide crucial information of human astrocyte (and glial in general) contributions to AD, as well as possibly describing ways to improve translatability.

## 5. Conclusions

As AD is a multifactorial disease, it is imperative to understand how different components contribute to disease pathogenesis in both humans and in rodent models. One major consideration is the differential contribution of each of the various cell types present in the CNS. Current models have helped us understand AD pathology, particularly in terms of neuronal dysfunction and synaptic loss, yet we still do not fully understand the role of astrocytes. To improve translatability, new models need to be developed that more accurately recapitulate the changes seen in human patients—and these initial steps have already been taken. Chimeric mice combine advantages from both hiPSCs and mouse models, while the MODEL-AD consortium has the potential to provide addition rodent models that incorporate complex and mixed background strains that could help our understanding of the disease and find ways to cure it. The new models provide a vast range of opportunities to design novel approaches to understand AD. The next decade will prove to be exciting in the field of neurodegeneration, including new insights into the role of astrocytes. How do astrocytes become reactive in AD, and what are the exact molecular mechanisms? Are such mechanisms preserved between different brain regions? How many different reactive states are there? If multiple reactive states are present, which ones play protective roles and which ones contribute to the pathology? What are the differences between mouse and human astrocytes under normal or pathological conditions? We are beginning to understand the complexity and context-dependent nature of heterogeneous astrocyte responses in many disease and infection models.

## Figures and Tables

**Table 1:** Reported changes in the GFAP, AQP4, complement pathway, and cytokines in astrocytes from APP or PSEN mouse models. All comparisons are versus age-matched, wild-type mice, unless otherwise mentioned. For more information on the genetic background models see Table 2. For a full overview in APP and/or PSEN mouse models see Supplementary Table S1. For an overview of astrocyte responses in Tau mouse models see Supplementary Table S2.

Finding	Method	Age	Brain Area	References
Tg(APPswe/PSEN1dE9) (also known as 2xTg, 2xTg-AD, APP/PS1)				
↑ GFAP protein (including staining intensity) (for male vs. female see [71,92])	WB, IHC	6–19 mo 3 mo (HPC)	CTX, HPC STR, CB (19 mo)	[26,72,73,74,75,76,93]
↑ GFAP+ cell density	IHC	6, 12–14, 23–28 mo ? 6, 12–14 mo 3 mo 16 mo	CTX HPC DG CA3	[26,73,79,85]
↓ GFAP+ cell density	IHC	24 mo	HPC	[80]
NC GFAP+ cell density	IHC	5–9 mo	CTX layers II/III	[81]
↑ GFAP+ area in 8–12 mo	IHC	2–6, 8–12 mo	FtC, HPC	[77]
↓ GFAP+ area	IHC	24 mo	HPC	[80]
NC GFAP+ cells/blood vessel	IHC	6, 12–14, 23–28 mo	HPC	[79]
NC A1, A2, pan-reactive genes 235 genes differentially expressed [↑ Cytokines/Chemokines (*Il1β*, *Ccl2*, *Ccl4*, *Ccl6)*, Complement (*C1qa*, *C1qb*, *C1qc*, *C4b*)]	RNA-seq of FACS-isolated astrocytes	9 mo	HPC	[94]
No uptake of Methoxy-X04+ amyloid fibrils by astrocytes	FACS	9 mo	HPC	[94]
Fibrillar Aβ not engulfed by GFAP+ cells	IHC	3, 6, 9, 12 mo	CTX, HPC	[73]
Observation: hypertrophic astrocytes close to plaques, atrophic distant to plaques	IHC	24 mo	CTX, HPC	[71,73,77,79,80]
Tg(APPSwLon/PSEN1*M146L)				
GFAP+ cells engulf APP+ dystrophic neurites	IHC, EM	4, 6, 12 mo	HPC	[95]
Tg(PDGFB-APPSwInd) (also known as hAPP-J20, APP/J20, J20)				
↑ GFAP+ area from 12–29 mo	IHC	3, 9, 12–16, 29 mo	CTX	[96]
↓ GFAP+ surface/volume per cell	IHC	5 mo	HPC	[97]
NC GFAP+ cell surface and volume	IHC	5 mo	HPC	[97]
Observations: Vascular amyloidosis can partially or fully displace astrocyte endfeet from vessels	IHC, EM	27 mo	CTX	[96]
Tg(Thy1-APPSw/Prnp-PSEN2*N141I) (also known as PS2APP)				
↑GFAP+ area	IHC	6 mo	HPC	[33]
↑classical components (↑ *C1q* in RNA-seq, but not NC fluorescence intensity in IHC) NC *C3* expression ↑A1-specific and pan-reactive genes	RNA-seq of FACS-isolated astrocytes (validated with IHC)	7, 11.5, 13 mo	HPC	[33]
Observation: C3 mostly associates with astrocytes	IHC	6 mo	HPC	[33]
Tg(Thy1-APPSweArc)B (also known as Tg-Arc/Swe, TgArcSwe)				
↑ AQP4 in 9 mo, NC AQP4 at 12 mo	WB	9, 12 mo	FtC	[98]
↑ AQP4 staining intensity	IHC	4, 16 mo	CTX	[40]
Observation: Loss of AQP4 polarization in astrocytes close to Aβ plaques	IHC	8–16 mo	*	[98]
Observations: 3 senile plaque stages characterized: (1) GFAP+/AQP4-; (2) GFAP+/AQP4+; (3) GFAP-/AQP4- 2 types of astrocytes related to Aβ observed: (1) ↑ AQP4, (2) less ↑ AQP4, rich in mitochondria, microvesicles	IHC, EM	8, 12, 16 mo	CTX	[40]
Tg(APPSwe)2576 (also known as APPSw, APPswe, Tg2576)				
GFAP colocalization with human APP	IHC	3, 18 mo	CTX, CC	[99]
GFAP associates with pyroglutamate-modified Aβ peptides	IHC	*	*	[100]
NC Astrocyte end-feet (assessed by GFAP)	IHC	12 mo	FtC, HPC	[101]
NC AQP4 associated w/vessels	IHC	12 mo	FtC, HPC	[101]
Tg(Thy1-APPArc)M8 (also known as TgAPParc, Thy1.2-hAPParc)				
Observations: Loss of endfeet contact with vessels in plaques (6–13 mo). Maintained GFAP-vessel interaction at non-CAA vessels 16–22 mo	IHC	6, 9–13, 16–22 mo	CTX	[102]
Tg(PRNP-APPSweInd)8 (also known as TgCRND8, Tg19959)				
↑ GFAP+ cell density around plaques	IHC	3, 6 mo	CA1	[103]
↑ GFAP signal intensity	IHC	3, 6 mo	CA1	[103]
↑ GFAP+ branch length	IHC	3, 6 mo	CA1	[103]
NC GFAP signal intensity	IHC	3, 6 mo	CA3	[103]
NC GFAP+ cell density around plaques	IHC	3, 6 mo	CA3	[103]
NC GFAP+ branch length	IHC	3, 6 mo	CA3	[103]
Tg(Thy1-APPSwDutIowa) (also known as TgSwDI)				
↓ astrocyte end feet number	IHC (using GFAP)	12 mo	FtC, HPC	[101]
↓ AQP4 vessel coverage	IHC (using GFAP)	12 mo	FtC, HPC	[101]

Abbreviations: ↑ = upregulation; ↓ = downregulation; NC = no significant change; ? = contradicting data; * = missing data; Aβ = amyloid beta; APP = amyloid precursor protein; CB = cerebellum; CA = Cornu Ammonis; CTX = cortex; DG = dentate gyrus; EM = electron microscopy; EC = entorhinal cortex; FACS = fluorescence-activated cell sorting; FtC = frontal cortex; HPC = hippocampus; IHC = immunohistochemistry; mo = months old; NC = no significant change; STR = striatum; WB = Western blot; WT = wild-type. For additional mouse genome synonyms we recommend referring to www.informatics.jax.org.

**Table 2:** Genetic background of FAD mouse models discussed in the review. Late-onset Alzheimer’s disease models are not included here, as the genetic manipulation is indicated in the model’s name (e.g., human GFAP-APOε4 = APOε allele 4 driven by the GFAP promoter). APP695, 751, and 770 refer to different APP isoforms generated by alternative splicing of exons 7 and 8. * All mice are transgenic, except for 3xTg, where PSEN1 was knocked-in. For additional information on these models and the models in Supplementary Tables S1–S3, please visit (https://www.alzforum.org/research-models/alzheimers-disease). For ease of reading, we will refer to the shortened model name (left hand column) throughout the review. Addition synonyms can be found at www.informatics.jax.org.

Model	Gene(s)	Mutation(s)	Promoter	References
mThy1-hAPP751	*APP*	*APP751*; Swedish (K670N/M671L) and London (V717I)	*mThy1*	[106]
Tg2576	*APP*	*APP695*; Swedish (K670N/M671L)	Hamster *Prnp*	[107]
TgAPParc	*APP*	*APP695*; Arctic (E693G)	*mThy1.2*	[108]
TgArcSwe	*APP*	*APP695*; Arctic (E693G) and Swedish (KM670/671NL)	*mThy1*	[109]
TgSwDI	*APP*	*APP770*; Swedish (K670N/M671L), Dutch (E693Q) and Iowa (D694N)	*mThy1*	[110]
5xFAD	*APP, PSEN1*	*APP695*: Swedish (K670N/M671L), Florida (I716V), London (V717I) *PSEN1*: M146L and L286V	*mThy1*	[111]
APPPS1	*APP, PSEN1*	*APP751*: Swedish (K670N/M671L) *PSEN1*: L166P	*mThy1.2*	[112]
APPswe/PSEN1dE9	*APP, PSEN1*	*APP695*: Swedish (K670N/M671L) *PSEN1*: exon 9 removed	m*Prnp*	[113,114]
APP_SWE/LON_/PSEN1^M146L^	*APP, PSEN1*	*APP*751: Swedish (K670N/M671L) and London (V717I) *PSEN1*: M146L	*mThy1 (APP)* *HMG (PSEN1)*	[115]
PSEN2APP	*APP, PSEN2*	*APP751*: Swedish (K670N/M671L) *PSEN2*: N141I	*mThy1.2* *mPrnp*	[116]
PS19	*MAPT*	*MAPT* (1N4R): P301S	*mPrnp*	[117]
Tau^R406W^	*MAPT*	*MAPT* (2N4R): R406W, contains myc and FLAG tags at N-and C-terminal ends, respectively	*CamkII*	[118]
Tau^P301L^	*MAPT*	*MAPT* (2N4R): Tau-4R/2N isoform; P301L	*mThy1*	[118]
rTg4510	*MAPT*	*MAPT* (0N4R): P301L	tetO *CaMKIIα*-tTA	[119,120]
rTgTauEC	*MAPT*	*MAPT* (0N4R): P301L	tetO *Neuropsin*-tTA	[121,122]
3xTg	*APP, PSEN1, MAPT*	*APP751*: Swedish (K670N/M671L) *PSEN1*: M146V *MAPT* (0N4R): P301L	mThy1 *	[57]

## Supplementary Materials

The following are available online at https://www.mdpi.com/2073-4409/9/11/2415/s1, Overview of literature search parameters. Table S1: Main astrocytic findings from APP and PSEN mouse models. Table S2: Main astrocytic findings from Tau mouse models: MAPT and APP/MAPT/PSEN (3xTg). Table S3: Main astrocytic findings from LOAD and LOAD/FAD mouse models. Supplementary References.
