# Supplementary material for: An Overview of Astrocyte Responses in Genetically Induced Alzheimer’s Disease Mouse Models
Source: Cells. 2020 Nov 4;9(11):2415. doi: 10.3390/cells9112415 (PMC7694249; doi:10.3390/cells9112415)
Supplement: Supplementary file 1 [file cells-09-02415-s001.pdf]

SUPPLEMENTAL MATERIAL

1. Overview of literature search parameters.

2. Supplementary Table S1. Main astrocytic findings from amyloid-based mouse models.

3. Supplementary Table S2. Main astrocytic findings from Tau-based mouse models.

4. Supplementary Table S3. Main astrocytic findings from LOAD and LOAD/FAD mouse models

5. Supplementary References – additional references for material only included in Supplementary Tables 1-3.

Overview of literature search parameters.

The PubMed search engine (https://pubmed.ncbi.nlm.nih.gov/) was used for the literature search. These keywords were used as Title/Abstract search terms for in all searches: “Alzheimer's” OR “Alzheimer”, “astrocytes” OR “astrocyte” OR “astroglia” OR “astrogliosis”, “mouse” OR “mice”. Additional keywords were used depending on the genetic modification in the mouse models: for APP, “amyloid” OR “amyloid-beta” OR “Abeta” OR “Aβ”; for Tau, “Tau” OR “MAPT”; for ApoE, “ApoE” OR “Apolipoprotein” OR “Apolipoprotein-E”; for TREM2, “TREM2”. The literature was restricted to mouse models relevant to the purpose of the review, meaning only studies in transgenic/knock-in mice without additional genetic manipulation other than mutations inducing the AD phenotype in mice. For instance, specific protein KO (such as complement component proteins) studies were excluded as well as studies using drugs. Due to the purpose of the review and limited space models with chemical induction of AD (i.e. heavy metals, okadaic acid) or injection of Aβ or Tau in the mice, namely non-genetically induced AD mouse models, were also excluded. Furthermore, It is important to note that the cited papers could include in vitro work, which were excluded due to the purpose of the review. For acquiring the total number of studies in FAD and LOAD transgenic mouse models, astrocyte-related keywords were removed.

Supplementary Table S1. Main astrocytic findings from amyloid-based mouse models. All comparisons are versus age-matched wild-type mice unless otherwise mentioned. Findings included in **Table 1** are not presented in this table. Abbreviations: ↑ = upregulation; ↓ = downregulation; NC = no significant change; ? = contradicting data * = missing data; Aβ = Amyloid Beta, APP = Amyloid Precursor Protein, BS = Brain Stem, CB = Cerebellum, CA = cornu ammonis, CTX = Cortex, DG = Dentate Gyrus, EC = Entorhinal Cortex, ELISA = Enzyme-linked Immunosorbent Assay, EtBr = Ethidium Bromide, FACS = Fluorescence-activated Cell Sorter, FLIM = Fluorescence Lifetime Imaging Microscopy, FRAP = Fluorescence Recovery after Photobleaching, FtC = Frontal Cortex, GJ = Gap Junctions, HPC = Hippocampus, IHC = Immunohistochemistry, ISH = *In Situ* Hybridization, iTRAQ = Isobaric Tags for Relative and Absolute Quantitation, LM = Laconosum Moleculare, MX04 = Methoxy-X04, mo = months old, ParC = Parietal Cortex, PET = Positron Emission Tomography, qPCR = Quantitative Polymerase Chain Reaction, RGCL = Retinal Ganglion Cell Layer, SC = Spinal Cord, SO = Stratum Oriens, SP = Stratum Pyramidale, SR = Stratum Radiatum, STR = Striatum, TH = Thalamus, Tg = Transgenic mice, vs = versus (comparison), WB = Western Blot, WT = Wild Type, YFP = Yellow Fluorescent Protein, $[Ca^{2+}]_i$ = intracellular calcium concentration, ($[^{11}C]$DED = 11C-Deuterium-l-Deprenyl). For additional synonyms for individual mouse models we recommend referring to www.informatics.jax.org.

Finding	Method	Age	Brain Area	References
Tg(APPswe/PSEN1dE9) (also known as 2xTg, 2xTg-AD, APP/PS1)				
↑ GABA release, signal intensity	Microdialysis, IHC	10mo (male)	HPC	[56]
↑ MAOB activity	Enzyme Assay	10mo (female)	DG only	[56]
↑$[Ca^{2+}]_i$	IHC (Fluo-4 AM)	9mo	HPC	[222]
↑ Connexin EtBr uptake in 9mo (close and far from plaques)	EtBr uptake	2, 9mo	HPC	[222]
↑ Pannexin EtBr uptake (close to plaques) in 9mo	EtBr uptake	2, 9mo	HPC	[222]
Astrocyte gap junction communication maintained in Tg mice	FRAP (SR101 dye)	9mo	HPC	[222]
807 upregulated (top 5: *Cst7*, *Ccl4*, *Il1b*, *Clec7a*, *Tyrobp*)<br>571 downregulated genes (top 5: *Hes5*, *Fam123a*, *Gpd1*, *Igfbp2*, *Ppp1ar3g*)	Microarray of FACS-isolated astrocytes	15mo	CTX	[223]
↑ *Vim*, *Clec7a* in plaque vs non-plaque areas<br>NC *Aif*, *Tyrobp*, *Cst7*, *Hes5*, *Glul*, *Fgfr3*, *Slc1a3* in plaque vs non-plaque areas	Microarray of selected genes using laser micro-dissected tissue (plaque vs non-plaque areas)	9mo	CTX	[223]
33 genes of all age-upregulated genes had increased expression in 12mo Tg and WT mice were identified (top 5: *Cyb5r2*, *C4b*, *Chil1*, *Bdkrb2*, *Rnase4)*<br>53 genes of all age-downregulated genes had decreased expression in 12mo Tg and WT mice were identified (top 5: *Ret*, *Rflnb*, *Ankrd52*, *Abhd2*, *Abcc6*)	RNA-seq of FACS-isolated astrocytes (validation through qPCR)	2, 4, 6, 9, 12mo	Total	[224]
↑$[Ca^{2+}]_i$<br>Resting $[Ca^{2+}]_i$ did not depend on MX04+ plaque proximity<br>$[Ca^{2+}]_i$ transient amplitude did not depend on MX04+ plaque proximity<br>NC resting $[Ca^{2+}]_i$ between spontaneously active versus inactive astrocytes	FLIM assay using OGB dye in SR-101+ astrocytes	6-8mo	CTX	[225]
↑ spontaneously active astrocytes at 6-8mo Tg vs 3-3.5 Tg and 6-8mo WT mice	FLIM assay using OGB dye in SR-101+ astrocytes	3-3.5, 6-8mo	CTX	[225]

↑ astrocyte correlated activity (astrocyte pairs that were synchronously active)<br>Significant correlated activity of astrocyte pairs up to 200μm in Tg vs 50μm in WT	FLIM assay using OGB dye in SR-101+ astrocytes	6-8mo	CTX	[225]
↑ $[Ca^{2+}]_i$ transient amplitude during intracellular calcium wave event in Tg vs non-wave event in Tg and WT mice	FLIM assay using OGB dye in SR-101+ astrocytes, time-lapse imaging,	3-3.5, 6-8mo	CTX	[225]
Observation: intercellular Ca^{2+} waves seen in Tg mice were absent in WT mice	FLIM assay using OGB dye in SR-101+ astrocytes	6-8mo	CTX	[225]
GFAP colocalization with TRPA1	IHC	8mo (not 3mo)	CTX, HPC	[226]
GFAP colocalization with TSPO (↑ GFAP+ cells)	IHC	3mo	HPC	[26,58]
GFAP colocalization with LRP1 (↓ LRP1+ area)	IHC	12mo	CTX, HPC	[134]
GFAP colocalization with mGLUR5 around plaques	IHC	6, 16mo	CTX	[227]
GFAP colocalization with PEA15 (↑ signal intensity)	IHC	5mo	CTX	[228]
GFAP colocalization with NPD52	IHC	12mo	CTX, HPC	[229]
GFAP colocalization with TNF-α?, IL-6?, IL-1β?	IHC	8-10mo	CTX, HPC	[55,64]
GFAP colocalization with CPE, SGIII (in humans)	IHC	9mo (male)	CTX, HPC, TH	[230]
GFAP colocalization with Platelets	IHC	14mo	CTX, HPC	[231]
GFAP colocalization with SMO, GLI1 (↑signal intensity in GFAP+ cells)	IHC	15mo	EC, DG, CA1	[232]
GFAP colocalization with C3 (↑signal intensity in GFAP+ cells)	IHC	18mo	HPC	[57]
GFAP colocalization with mFRP1,2, RAGE	IHC	12mo	FtC	[233]
GFAP colocalization with KAT2 (↑ GFAP+ cells)	IHC	12mo	CTX, DG	[234]
GFAP colocalization with SOX2 (↓ GFAP+ cells)	IHC	12mo	CTX, DG	[234]
GFAP colocalization with HO-1,2	IHC	6mo	HPC	[235]
GFAP colocalization with A2A Receptor	IHC	16.5mo	HPC	[236]
GFAP colocalization with MIZ1	IHC	7mo	CTX, HPC	[237]
GFAP colocalization with CEBPδ	IHC	?	CTX	[238]
GFAP does not colocalize with BMP6	IHC	20mo	HPC	[239]
GFAP does not colocalize with Beclin1	IHC	12mo	CTX	[240]
GFAP does not colocalize with CD36, RAGE, NEP, iNOS,	IHC	3-12mo	CTX, HPC	[55]
GFAP does not colocalize with IL-4β, TGF-β, YM-1, Arg1	IHC	3-12mo	CTX, HPC	[55]
GFAP does not colocalize with BrdU	3 days or 4 weeks post-injection	15mo	CTX	[94]
↓ ABCA1	WB	11mo (male)	Total	[241]
↑ STI1	WB	9, 12mo	Total	[242]
Observation: Loss of AQP4 polarization	IHC	6.5, 12mo	CTX, HPC	[134]
Observation: Astrocytes do not migrate towards plaques	IHC, spatial analysis using a pair-correlation function	5-9mo	CTX II/III	[63]

Tg(APPSwFlLon/PSEN1*M146L*L286V)6799 (also known as 5xFAD, Tg-5xFAD, Tg6799)				
↑ GFAP signal intensity	IHC<br>WB<br>WB	4.5, 9mo<br>8mo<br>12mo	Whole brain<br>CA1, DG<br>CTX	[243,244]<br>[245]<br>[246]
↑ GFAP signal intensity (only values, no statistics)	IHC	2, 4, 9, 12mo	FtC, ParC, EC, DG	[244]
↑ *Gfap*	qPCR	4, 6mo	HPC	[247]
↓ AQP4+ & Lama2+ cells	IHC	6mo	CTX	[201]
↑ ABCC1	WB	1.5-12mo	CTX	[248]
↓ TSP1	WB	6, 9mo	HPC	[249]
↑ *Il1β*, *Tnf*	qPCR	2, 4, 6mo	HPC	[247,250]
No global protein SUMOylation changes	WB	6mo	Total	[251]
GFAP colocalization with C1qa (↑ *C1qa*)	IHC (qPCR)	14mo (6-12mo)	CTX	[246]
GFAP colocalization with p16, GS	IHC	18mo	CA1	[252]
GFAP colocalization with ΔCaN (↑GFAP & ΔCaN)	IHC (WB)	8mo	CA1, DG	[245]
GFAP colocalization with NFAT	IHC	8mo	CA1, DG	[245]
GFAP colocalization with MMP2 (↑MMP2 in 4mo)	IHC (WB)	2, 4, 6mo	HPC	[247,250]
GFAP colocalization with MMP9 (↓MMP9 in 2mo, ↑ in 4mo)	IHC (WB)	2, 4, 6mo	HPC	[247]
GFAP colocalization with OLFR110/111 (↑*Olfr110/111* in 9, 12mo)	IHC (qPCR)	4, 6, 9, 12mo	CTX, HPC	[253]
GFAP colocalization with OLFRR544	IHC (qPCR)	4, 6, 9, 12mo	CTX, HPC	[253]
GFAP colocalization with APP (↑ colocalization in 10mo vs 2mo)	IHC	2, 10mo	CA1-3, DG	[254]
GFAP does not colocalize with TSPO	IHC	6mo (female)	CTX	[255]
GFAP does not colocalize with GRP78, CHOP	IHC	7mo	FtC	[256]
Observation: GFAP surrounds Aβ and ceramide	IHC	10mo	?	[65]
Observation: Morphological changes of GFAP+ astrocyte endfeet	IHC	4.5, 9mo	?	[243]
Observation: Diffuse AQP4 and LAMA2 signal in Tg mice vs WT	IHC	6mo	CTX	[201]
Tg(APPSwe)2576 (also known as APPSw, APPswe,Tg2576)				
↑ GFAP signal intensity	IHC	6mo<br>12mo	CA1 (SLM)<br>HPC, CB	[257]<br>[257]
↑ GFAP+ cell density	IHC	12mo vs WT,<br>18-24mo vs 6mo Tg	FtC, HPC<br>HPC	[72]<br>[258]
↑ GFAP+ cell density close to plaques	IHC	19-29mo	CTX	[259]
↑ *Gfap*	qPCR, autoradiography	12-18mo	CTX, HPC	[260,261]
↑ *Gfap* close vs far from plaques	qPCR, autoradiography	12-18mo	CTX, HPC	[260,261]
↑ S100β+ area	IHC	12mo	FtC, HPC	[72]
↑ $[^{11}C]$DED binding	PET	6mo vs WT & vs 8-16, 18-24mo	CTX, HPC	[258]

↑ MAOB Activity (females only)	Enzyme Assay	18mo (male and female separately)	CTX, HPC	[102]
Possible relation between ↑ GFAP+ area and ↑ GABA release (females)	IHC, Spectroscopy	18mo (male and female separately)	CTX, HPC	[102]
NC Cx43	WB	12mo	FtC, HPC	[72]
NC KIR4.1, BK (K^+ channels)	WB, qPCR	12mo	FtC, HPC	[72]
↑ *S100β*, *Cx43*	qPCR	12mo	FtC, HPC	[72]
↓ TSP1	WB	12mo	CTX	[249]
GFAP colocalization with Kv3.4 (↑Kv3.4 signal intensity)	IHC	6mo	HPC, CB	[257]
GFAP colocalization with Hepcidin (↓Hepcidin)	IHC (WB)	10mo (6mo)	CTX, HPC	[262]
GFAP colocalization with PEBP only around plaques	IHC	12, 18mo	CTX	[263]
GFAP colocalization with TRH	IHC	?	?	[71]
GFAP colocalization with Leptin Receptor (↑GFAP+ cells in 15mo)	IHC (laser capture, WB)	7, 15mo	CA1	[264]
GFAP colocalization with MMP2, MMP9 (↑GFAP+ cells close to plaques)	IHC	12-15mo	Total	[260]
GFAP colocalization with MAOB	IHC	12, 18mo	CTX, HPC	[102]
GFAP colocalization with Nitrotyrosine (around plaques & vessels)	IHC	13, 16.5mo	CTX	[265]
GFAP colocalization with RAC	IHC	18mo	HPC	[266]
GFAP colocalization with Metallothionein-1 & 3	IHC	14-18mo	CTX?	[261]
GFAP colocalization with APOE, APOER2 (around plaques)	IHC	18-15mo	CTX	[267,268]
GFAP colocalization with IL-12, IL-6	IHC	17, 18mo	CTX, HPC	[92,269]
GFAP colocalization with BACE1 (around plaques)	IHC	17mo	ParC	[270]
GFAP colocalization with ?tPA	IHC	15-20mo	CTX?	[271]
GFAP colocalization with Caveolin-3	IHC	14-29mo	?	[272]
GFAP colocalization with Neprilysin (↑Neprilysin signal intensity in GFAP+ cells)	IHC	22mo	ParC	[273]
GFAP colocalization with Cystatin-2	IHC	3-16mo	CTX, HPC	[274]
GFAP associates with HTT	IHC	15-32mo	CTX, HPC	[275]
GFAP does not colocalize with Synaptotagmin 4	IHC	19-29mo	CTX	[259]
Observation: Astrocytosis (↑ [^{11}C]DED binding) before plaque formation.	IHC	6mo	CTX, HPC	[258]
Observation: Majority of GFAP+ cells are atrophic in 6mo. Hypertrophic astrocytes near plaques in 18-24mo.	IHC	6, 18-24mo	CTX, HPC	[258]
Observation: GFAP+ reactive astrocytes around diffuse & fibrillary plaques	IHC	13-19mo	CTX	[92,267,276]

Observation: ↑ TGF-β1, TGF-β3, IL-6, IL-10 in few astrocytes (no cellular staining)	IHC	13, 16, 19mo	CTX	[276]
Observation: ↑ MDA around astrocytes	IHC	15-17mo	CTX	[277]
Tg(PDGFB-APPSwInd) (also known as hAPP-J20, APP/J20, J20)				
NC GFAP+ cell density	IHC	3mo	CTX	[278]
NC GFAP+ area	IHC	3, 6, 9, 12mo	CTX	[278]
↓ GFAP signal intensity in 1mo	IHC	1, 3. 5, 7, 18mo	HPC	[279]
↑ GFAP+ area in 9mo	IHC	3, 6, 9, 12mo	HPC	[278]
↑ GFAP	WB	2.5-3mo	HPC	[280]
↑ GFAP+ cell density	IHC	9, 15mo	Hilus	[68,281]
↓ GFAP+ cell density	IHC	9, 15mo	SR	[68,281]
NC GFAP+ cell density	IHC	9, 15mo	SR	[68,281]
↑ GFAP+ cell surface & volume close to plaques	IHC	9, 15mo	SR	[281]
↓ GFAP surface/volume per cell close to plaques	IHC	9, 15mo	SR	[281]
NC Vimentin+ & S100β cell density	IHC	3mo	CTX	[125]
NC GFAP, Vimentin, ALDH1L1, Cx43, GS & GLT-1	WB	3mo	CTX, HPC	[125]
↓ AQP4+ area from 3 to 16mo	IHC	3, 9, 12-16, 29mo	CTX	[67]
131 genes significantly altered: ↑levels of oxidative stress/cell death proteins, ↑ protein, degradation pathway, ↑ demyelination, altered inflammation, Aβ deposition and cell polarity (↑COL1A2, UCHL1; ↓SLC1A2, SIRT2, PRDX6, GSTM1, LSAMP, SNCB, S100B)	iTRAQ proteomics (WB, qPCR)	2.5-3mo	HPC	[280]
GFAP colocalization with_LC3 (↓GFAP+ cells in 20mo vs 15mo in Tg)	IHC	9, 15, 20mo	SR	[281]
GFAP colocalization with_mGlu2/3R (↓GFAP+/mGlu2/3R+ cells in 14mo)	IHC	5, 14mo	HPC	[282]
GFAP colocalization with_A2A Receptor (↑A2A signal intensity in GFAP+ cells	IHC	17mo	HPC	[236]
Observation: Reactive astrocytes located around Aβ plaques	IHC	from 9mo	HPC, CTX	[278]
Tg(Thy1-APP) (also known as APP23)				
↓ ABCA1	WB	13mo	Total	[283]
NC APOE	WB	13mo	Total	[283]
S100A6, S100A8, S100B localize with Aβ plaques in 15mo	IHC	3, 15mo	CTX, HPC	[284]
↑ S100A8, S100B CTX in 15mo	WB	3, 15mo	CTX, CB	[284]
GFAP colocalization with Transglutaminase	IHC	24-27mo	?	[285]
GFAP colocalization with PBR	IHC	20mo	HPC	[286]
GFAP colocalization with GDNF (around plaques)	IHC	20mo	HPC	[286]
GFAP colocalization with BDNF (only around plaques)	IHC	21mo	CTX	[287]
GFAP colocalization with CyclinE, D1, B1 & CDK4	IHC	21.5mo (female)	CTX	[288]

GFAP does <u>not</u> colocalize with CDKIs	IHC	21.5mo (female)	CTX	[288]
Observation: Astrocytes express endothelial NOS and/or inducible NOS	IHC	18mo	CTX, HPC	[289]
Tg(APPArcSwe) (also known as Tg-ArcSwe)				
↑ GFAP	ELISA	8, 12, 16mo	Total	[290]
↑ GFAP signal intensity	IHC	8, 12, 16mo vs 4mo	CTX	[40]
↑ [^{11}C]DED binding in 16mo TH	PET	8, 16mo	CTX, HPC, STR, TH	[290]
GFAP does <u>not</u> colocalize with MAOB	IHC	8, 12, 16mo	HPC	[290]
Tg(PRNP-APPSweInd)8 (also known as TgCRND8, Tg19959)				
↑ TNF-α+ & IL-1β+ cell density in 6mo	IHC	3, 6mo	CA1, CA3	[74]
↑ NeuN/GFAP/Iba1 colocalization in 6mo	IHC	3, 6mo	CA1, CA3	[74]
GFAP colocalization with PEA-15 (around plaques, ↑PEA-15 in 6mo)	IHC, Mass Spectrometry	3, 6mo	CTX	[291]
GFAP colocalization with IL-6, IL-1β	IHC	9mo	CTX	[292]
GFAP colocalization with phospho-p38	IHC	7mo	HPC	[293]
Observation: GFAP+ cells surround compact & diffuse plaques	IHC	9-20 weeks old	CTX, Subiculum	[294]
Observation: Characterized reactive glial nets (microglia and astrocytes forming 3D structures in AD) in mouse and human	IHC	9mo	CTX	[292]
Tg(Thy1-APPSwDutIowa) (also known as TgSwDI)				
↑ GFAP+ cell density in 24mo Tg vs 24mo WT & 6mo Tg	IHC	6, 24mo	CTX	[295]
↑ GFAP+ cell density in 12mo	IHC	6, 24mo	CTX	[295]
↑ GFAP+ cell density in 3mo Subiculum	IHC	3, 12mo	FtC, HPC, TH, Subiculum	[72,296]
↑ *Gfap* in CA1, Subiculum	Autoradiography (ISH)	5, 12, 18mo (males)	CTX, DG, CA1, Subiculum, Thalamus, LM	[261]
↑ *Gfap* in 18mo CTX & TH	Autoradiography (ISH)	5, 12, 18mo (males)	CTX, DG, CA1, Subiculum, Thalamus, LM	[261]
↑ S100β+ area and mRNA	IHC, qPCR	12mo	FtC, HPC	[72]
↓ CX43, KIR4.1, BK (K+ channels)	WB, qPCR	12mo	FtC, HPC	[72]
↑ AQP4 vessel coverage in 24mo vs WT	IHC (using GFAP)	6, 24mo	CTX	[295]
↓ AQP4 vessel coverage in 24mo vs 6mo Tg	IHC (using GFAP)	6, 24mo	CTX	[295]
GFAP does <u>not</u> colocalize with C1q, C3, C4	IHC	12mo	TH	[297]
Tg(APPSwLon/PSEN1*M146L)				
↑ GFAP+ area (plateau reached in 9mo)	IHC	4, 6, 12mo	HPC	[66]

↑ BLBP+ area	IHC	4, 6, 12mo	HPC	[66]
NC BLBP+ cell density	IHC	6mo	Hilus	[298]
GFAP colocalization with S100A6	IHC	2.5-10mo	CTX	[299]
Observations: AQP4 and EAAT2 surround dystrophic neurites in astrocytes	IHC, Electron microscopy	4, 6, 12mo	HPC	[66]
Tg(tetO-APPSwInd) (also known as tet-APPswe/ind)				
NC YFP transfer through gap junctions (opposite to *in vitro* experiments)	*Ex vivo* slice, YFP injection, dye transfer	8.5-14mo	CTX, HPC, Inferior Colliculus	[300]
GFAP colocalization with C3 (↑C3 signal intensity per GFAP+ cell)	IHC	8mo	HPC	[57]
Tg(Thy1-APPLon)2 (also known as APP/V717I)				
↑ GFAP+ cell density in 16mo Tg vs 3mo WT and 16mo WT	IHC	3, 16mo	CTX, HPC	[301]
↑ GFAP+ cell density in 3mo Tg vs 3mo WT	IHC	3, 16mo	CTX, HPC	[301]
↑ *Gfap* in 16mo Tg vs 16mo WT	qPCR	3, 16mo	CTX, HPC	[301]
GFAP colocalization with iNOS (↑GFAP+ cell density 16mo Tg vs 16mo WT)	IHC	3, 16mo	CTX, HPC	[301]
Tg(APPSw/PSEN1*M146L) (also known as PS/APP, APP$_{K670N/M671L}$/PS1$_{M164L}$, Tg2576/PS1)				
↑ GFAP+ area (plateau reached in 9mo)	IHC	2, 3, 5, 9, 12mo	CTX, HPC	[302]
↑ GFAP in 11, 17mo	ELISA	3, 6, 11, 17mo	HPC	[303]
GFAP colocalization with SRCL1 (close to plaques and vessels)	IHC	9mo	CTX, HPC	[304]
GFAP does not colocalize with SRCL1 (far from plaques or in WT)	IHC	9mo	CTX, HPC	[304]
GFAP colocalization with COX2 around plaques (not in human)	IHC	7mo	CTX, HPC	[93]
Observation: First GFAP staining around plaques at FtC? GFAP becomes abundant in 7mo.	IHC	3mo	FtC	[93]
Observation: Patches of GFAP staining in HPC, CC	IHC	6mo	HPC, CC	[303]
Observation: Patches of GFAP staining in CTX	IHC	3mo	CTX	[303]
Observation: Atrophic astrocytes	IHC	18, 30mo	CTX	[93]
Tg(PDGF-APPIn) (also known as PDAPP(line109), APPInd, APP^{V717F}, hbeta-APP)				
Astrocytes form networks in Tg CTX	Biocytin dialysis & diffusion, IHC	21-28mo	CTX, HPC	[305]
NC number of astrocytes displaying passive or outward-rectifying currents	Patch clamping followed by hyperpolarization or depolarization	21-28mo	CTX, HPC	[305]
CTX: ↑ double-responsive astrocytes<br>HPC: ↑Kainate-only & Kainate- and D-aspartate- responsive astrocytes, ↓no-responsive astrocytes<br>HPC: cells close to plaques: 75% Kainate-only, 25% Kainate- and D-aspartate- responsive astrocytes	Electrophysiology in the presence of Kainate and/or D-aspartate	21-28mo	CTX, HPC	[305]

GFAP colocalization with membrane bound LRP at distal GFAP+ processes	IHC	9-15, 22mo	?	[306]
Tg(APPSw)40 (also known as APPswe(R1.40), APP$^{K670/M671}$, R1.40, R1.40-YAC)				
↓ GFAP signal intensity at 9mo<br>↑ GFAP signal intensity at 21mo	IHC	4, 21mo	HPC	[307]
App$^{tm3.1Tcs}$ (also known as APP^{NL-G-F}, APP NL-G-F Knock-in)				
207 genes upregulated vs WT mice<br>73 genes downregulated vs WT mice<br>54 of the significantly altered genes were common with human AD samples from temporal cortex (↑*C4a*, *C4b*, *Cd74*, *Ctss*, *Gfap*, *Phyhd1*, *S100b*, *Tf*, *Tgfbr2*, *Vim*)<br>3 of the significantly altered genes were common with human AD samples from frontal cortex (↑*C4a*, *C4b*, *Phyhd1*)<br>5 of the significantly altered genes were common with human AD samples from temporal and frontal cortex (↑*C4a*, *C4b*, *Phyhd1*)	Microarray from bulk tissue (validated by qPCR)	12mo	CTX	[308]
↑ *Gfap* and *S100β*; differences between sexes are present which are more profound in older mice	qRT-PCR	5, 7, 12mo (males vs females)	CTX	[308]
↑ GFAP signal intensity in 12mo vs WT (males & females)<br>↑ GFAP signal intensity in 7mo FtC vs WT (males only)	IHC	5, 7, 12mo (males vs females)	FtC, Temporal CTX	[308]
App$^{tm2.1Tcs}$ (also known as APP^{NL-F})				
↑ GFAP signal intensity in 9-18mo	IHC	1-3, 4-6, 9-18mo	CA1	[309]
GFAP colocalization with_P2Y1 (↑GFAP/P2Y1 colocalization)	IHC	9-18mo	CA1	[309]
Tg(Thy1-APPSw,Thy1-PSEN1*L166P)21 (also known as APPPS1, APP.PS1)				
↑ hyperactive (> 4 Ca^{2+} transients / minute) & ↓inactive astrocytes<br>↑ hyperactive <50μm from plaques vs >50μm<br>↑ inactive >50μm from plaques vs <50μm	IHC, electrophysiology	5-9mo	CTX (L1-3)	[39]
NC Ca^{2+} transient amplitude, duration	IHC, electrophysiology	5-9mo	CTX (L1-3)	[39]
↑ spontaneously active endfeet percentage / cell (spontaneous Ca^{2+} elevations occurring in astrocyte endfeet)	IHC (OGB-1)	5-9mo	CTX (L1-3)	[39]
NC Ca^{2+} transient amplitude, duration at endfeet	IHC, electrophysiology	5-9mo	CTX (L1-3)	[39]
↑ tdTomato+ astrocyte volume & area	IHC (*hGFAP*-Cre*ERT2*, *Rosa*-tdTomato	3, 6, 12mo	CTX	[310]
↑ tdTomato+ astrocyte process length & number of branches from 6mo	IHC (*hGFAP*-Cre*ERT2*, *Rosa*-tdTomato	3, 6, 12mo	CTX	[310]
↑ GFAP+/tdTomato+ of total tdTomato+ cells	IHC (*hGFAP*-Cre*ERT2*, *Rosa*-tdTomato	6mo	CTX	[310]

NC S100β+/tdTomato+ of total tdTomato+ cells	IHC (*hGFAP*-Cre*ERT2*, *Rosa*-tdTomato	6mo	CTX	[310]
↓ GS+/tdTomato+ of total tdTomato+ cells	IHC (*hGFAP*-Cre*ERT2*, *Rosa*-tdTomato	6mo	CTX	[310]
↑ GFAP+, S100β, GS+ area	IHC	6mo	CTX	[310]
tdTomato+ astrocytes do <u>not</u> colocalize with BrdU (tamoxifen 4 weeks prior to analysis, two BrdU injections daily for 8 days prior to analysis)	IHC (*hGFAP*-Cre*ERT2*, *Rosa*-tdTomato	6mo	CTX	[310]
↓ GS from 6mo	WB	3, 6, 12mo	CTX	[310]
↓ GS activity from 6mo	Enzyme catalyzed chromogenic reaction assay	3, 6, 12mo	CTX	[310]
218 genes upregulated, 107 genes downregulated belonged to 10 gene clusters (signal peptide, disulfide bond, glyco-protein, secreted, hydrolase, calcium, inflammatory, immunity, lectin, and chemotaxis)	RNA-seq of FACS-isolated astrocytes	3mo	CTX	[310]
Age-dependent ↑ of P2Y1R concentration (ng/ml) in Tg mice	ELISA	6mo	Total	[311]
GFAP colocalization P2Y1R near methoxy-X04+ Aβ plaques	IHC	6mo	CTX, HPC	[311]
Positive correlation of P2Y1R concentration with GFAP+ area	ELISA (P2Y1R), IHC (GFAP)	6mo	Total	[311]
↑ Ca^{2+} transient amplitude<br>NC full duration at half maximum (FDHM)<br>↑ fraction of spontaneously active astrocytes	Astrocyte-specific (*GfaABC1D*) fluorescent calcium indicator (GCaMP6f) expression via AAV infection	6mo	HPC	[311]
Tg(Thy1-AppDutch) (also known as APPDutch)				
SR101 colocalizes with NAPDH-FLIM	IHC	18-24mo	CTX	[312]
GFAP colocalization with NOXO1	IHC	18-24mo	CTX	[312]
Tg(Thy1-APPSweLon)41 (also known as APPSL, APP41, TASD41, mThy1-hAPP751)				
↑ GFAP+ area at 6, 9mo vs 3mo Tg and 6, 9, 12 mo WT	IHC	6, 9, 12mo	CTX	[313]
NC GFAP+ area	IHC	6, 9, 12mo	HPC	[313]
GFAP colocalization with $Ca_v1.2$	IHC	6, 12mo	CTX, HPC	[314]
GFAP colocalization with $Ca_v1.2$ (around plaques)	IHC	2, 4, 11mo	HPC	[315]
GFAP does <u>not</u> colocalize with Ca^{2+} channel β4 subunit	IHC	2, 4, 11mo	CTX, HPC	[315]
Observation: ↑ GFAP & $Ca_v1.2$ signal intensity	IHC	11mo	CTX	[315]
Tg(Thy1-APPArc)M8 (also known as TgAPParc, Thy1.2-hAPParc)				
↓ β-dystroglycan vessel coverage in 16-22mo	IHC	6, 9-13, 16-22mo	CTX, HPC	[73]
Observation: Retracted and swollen endfeet observed around plaques	IHC	6, 9-13, 16-22mo	CTX	[73]
Tg(Prnp-APPOsk) (also known as APP E693Δ-Tg (Osaka))				
Observation: GFAP staining from 18mo	IHC	8, 12, 18, 24mo	CTX, HPC, CB	[316]

Tg(Thy1-APPSw/Thy1-PSEN1*G384A) (also known as APPswe/PS1G93A, APPswexPS1G384A)				
↑ S100β+ astrocyte soma size in 4-11moTgvs 1-2mo Tg	IHC	1-2, 4-6, 9-11mo	CTX	[317]
↑ S100β+ astrocyte soma size in 4-6mo Tg vs 1-2, 9-11mo Tg	IHC	1-2, 4-6, 9-11mo	DG	[317]
↑ GABA signal intensity in astrocytes in 4-6mo Tg vs 1-2, 9-11mo Tg	IHC	1-2, 4-6, 9-11mo	CTX, DG	[317]
↑ S100β+ soma size of astrocytesclose to plaques vs far from plaques	IHC	1-2, 4-6, 9-11mo	CTX, DG	[317]
↑ GABA signal intensity of astrocytes close to plaques vs far from plaques	IHC	4-6mo	CTX	[317]
NC S100β soma size and GABA signal intensity results in cumulative frequency graphs	IHC	4-6mo	CTX	[317]
Tg(Thy1-PSEN1*G384A) (also known as $PSEN1^{G384A}$)				
↓ S100β+ astrocyte soma size	IHC	4-6mo	CTX	[317]
NC GABA signal intensity in astrocytes	IHC	4-6mo	CTX	[317]
NC astrocyte number with altered S100β+ soma size	IHC	4-6mo	CTX	[317]
↑ astrocyte number with ↑ GABA signal intensity	IHC	4-6mo	CTX	[317]

Supplementary Table S2. Main astrocytic findings from Tau-based mouse models. All comparisons are versus age-matched wild-type mice unless otherwise mentioned. Abbreviations: ↑ = upregulation; ↓ = downregulation; NC = no significant change; ? = contradicting data * = missing data; Aβ = Amyloid Beta, BS = Brain Stem, CB = Cerebellum, CA = cornu ammonis, CTX = Cortex, DG = Dentate Gyrusx, EC = Entorhinal Cortex, FACS = Fluorescence-activated Cell Sorter, FtC = Frontal Cortex, GJ = Gap Junctions, HPC = Hippocampus, IHC = Immunohistochemistry, LM = Laconosum Moleculare, mo = months old, ParC = Parietal Cortex, qPCR = Quantitative Polymerase Chain Reaction, RGCL = Retinal Ganglion Cell Layer, SC = Spinal Cord, SO = Stratum Oriens, SP = Stratum Pyramidale, SR = Stratum Radiatum, STR = Striatum, TH = Thalamus, Tg = Transgenic mice, vs = versus (comparison), WB = Western Blot, WT = Wild Type. For additional synonyms for individual mouse models we recommend referring to www.informatics.jax.org.

Finding	Method	Age	Brain Area	References
Tg(Prnp-MAPT*P301S)PS19 (also known as Tau P301S (PS19), P301S Tau, PS19Tg)				
↑ GFAP+ cells in 3, 5mo	IHC	2, 3, 5mo	CTX	[140]
↑ GFAP+ area in 9mo Tg & 13mo WT vs 3mo WT	IHC	3, 6, 9mo (Tg), 3, 13mo (WT)	DG, CA1, CA3	[141]
↑ classical components (↑*C1q* in RNA-seq and C1q signal intensity in IHC)<br>↑ A1-specific & pan-reactive genes	RNA-seq of FACS-isolated astrocytes (validated by IHC)	6mo (validated in 9mo)	HPC	[33]
GFAP colocalization with C3	IHC	6mo	HPC	[33]
GFAP colocalization with GDNF	IHC	9mo	HPC	[141]
GFAP does <u>not</u> colocalize with PBR	IHC	9mo	HPC	[141]
Observations: NFT distribution & density corresponded that of GFAP.<br>Most astrogliosis observed in WM where there is little tau pathology.	IHC	3, 6mo	EC, HPC, AM, SC, WM	[117]
Fgf14 $^{Tg(tetO-MAPT*P301L)4510}$ (also known as rTg4510, Tg4510, Tg(tauP301L)4510, rTgP301L)				
↑ GFAP+ area in 6, 9 and 12mo Tg<br>↑ GFAP+ area in 12mo Tg vs 6 and 9mo Tg	IHC	6, 9, 12mo	FtC	[318]
↑ GFAP+ area in 9 and 12mo Tg<br>↑ GFAP+ area in 12mo Tg vs 6 and 9mo Tg	IHC	6, 9, 12mo	HPC	[318]
Observation: increased GFAP signal intensity from 2.5mo which further increased up to 12mo	IHC	1.5, 2.5, 4, 6, 8, 12mo	CTX, HPC	[144]
Observation: Hypertrophic astrocytes with thick and fibrous processes are present in 9 and 13mo Tg mice. Swollen astrocytic processes surround degenerating neurons of 4 and 13mo Tg mice.	Electron Microscopy	4, 9, 13mo	Dorsal CTX	[319]
rTgTauEC (also known as neuropsin-tTA x FVB-Tg(tetO-tauP301L)4510)				
↑ GFAP+ cell density in 14, 24, 34mo vs WT	IHC	8, 14, 24, 34mo	EC	[142]
GFAP colocalization with PHF-1	IHC	24mo	ML (or EC?)	[122]
GFAP does <u>not</u> colocalize with hTau (MC1 antibody)	IHC	8, 14, 24, 34mo	EC	[142]
Observations: ’reactive’ astrocytes in Amygdala. No reactive astrocytes in other brain regions.	IHC	8, 14, 24, 34mo	EC	[142]

Tg(MAPT)8cP (also known as hTau, 8c)				
↑ GFAP signal intensity in 24mo	IHC	3, 11, 24mo	CTX	[143]
GFAP colocalization with Tau in 11, 24mo	IHC (T22)	3, 11, 24mo	CTX	[143]
Tg(Prnp-MAPT*R406W)21807 (also known as Tau R406W Tg, TgTau406W)				
Observations: Reactive astrocytes are present. These astrocytes do not express human Tau, but do express mouse Tau	IHC	10mo	CTX, HPC, Amygdala	[157]
GFAP-hTauWT (also known as GFAP-hTauP301L, GFAP/tauP301L Tg)				
↓ GLT1 in SC 12mo & SC, BS 24mo<br>↓ GLAST in SC 5mo & SC, BS 12,24mo<br>(for both genotypes vs non-Tg controls)	WB	5, 12, 24mo	CTX, BS, SC	[320]
Tg(Thy1-MAPT)183 (also known as P301L tau, pR5)				
GFAP colocalization with Tau in 11,24mo	IHC (T22)	3, 11, 24mo	CTX, Retina	[143]
Observation: Filamentous Tau in GFAP+ subpial astrocytes	Electron microscopy	?	SC	[159]
TTg(Thy1-MAPT)22 (also known as THY-Tau22)				
↑ GFAP protein levels	WB	6, 12mo	HPC	[321]
↑ ADK protein levels at 12mo	WB	6, 12mo	HPC	[321]
Tg(APPSwe,tauP301L)1Lfa Psen1^{tm1Mpm} (also known as 3xTg, 3xTg-AD)				
↑ GFAP protein levels in 18-26mo<br>NC GFAP protein levels in 12mo	WB	6, 12, 18-26mo	HPC	[25,160–162]
↑ GFAP+ area	IHC	12mo	HPC	[25]
↑ GFAP+ area in 5-20 & 30-40 weeks old	IHC	5-20, 30-40, 50-72<br>weeks old	RGCL	[163]
↑ GFAP in 22, 26mo Tg vs 3, 12mo Tg	WB	3, 12, 22, 26mo	Subiculum, CA1	[164]
↑ GFAP+ cell density in 28mo Tg vs 18mo Tg	IHC	11, 18, 20, 28mo	Subiculum, CA1	[164]
NC GFAP signal intensity	IHC	22-24mo (female)	CTX, CA1, CA3, DG	[322]
NC GFAP+ cell density	IHC	3, 9, 12, 18mo (male)	PFC	[323]
↑ GFAP+ cell density	IHC	18-26mo	Alveus	[161]
↓ GFAP+ cell surface & volume from 3mo in total<br>↓ GFAP+ cell surface & volume from 3mo in layers I-II, IV-V<br>↓ GFAP+ cell surface & volume from 12mo in layer III	IHC	3, 9, 12, 18mo (male)	PFC	[323]
↓ S100β, Cx43 & ↑AQP4 in 12mo vs 6mo in Tg and WT mice	WB	6, 12mo	HPC	[160]
↑ TNF-α in 12,22 mo Tg vs 3,26 mo Tg	WB	3, 12, 22, 26mo	Subiculum, CA1	[164]
↓ *Il6*, *Ifmb* in 3mo<br>↑ *Il6*, *Ifnβ* in 12mo	qPCR	3, 12mo	CTX, HPC	[165]

↓ GS+ cell density (DG in 12,18mo, CA1 in 18mo)<br>↓ GS optical density in 18mo<br>↓ GS+ cell density close vs far from plaques<br>↓ GS+ than GFAP+ cell density both close and far from plaques	IHC	9, 12, 18mo	DG, CA1	[324]
No accumulation of intracellular lactate under the presence of monocarboxylate transporter blocker (AR-C155858)	Infection with adeno-associated virus 2/9 carrying Laconic (lactate FRET nanosensor)	6mo	HPC	[325]
↓ Total and extracellular L-Serine<br>↓ Total and extracellular L-Serine	HPLC (total levels)<br>*In vivo* microdialysis and LC-MS/MS	6mo	HPC	[325]
↑% of thin/long needle-shaped/oblong cells in EC vs HPC<br>↓% of flat/stellate in EC vs HPC	IHC	7 days old	EC, HPC	[326]
80 genes upregulated vs WT mice<br>171 genes downregulated vs WT mice<br>14 of the significantly altered genes were common with human AD samples from temporal cortex (↓*Vegfa*)<br>1 of the significantly altered genes were common with human AD samples from frontal cortex<br>2 of the significantly altered genes were common with human AD samples from temporal and frontal cortex	Microarray from bulk tissue (validated by qPCR)	12mo	CTX	[308]
GFAP colocalization with GS (↑ GFAP+ cell density)	IHC	9, 12, 18-26mo	Alveus, SO, CA1 DG	[161]
GFAP colocalization with KIR6.2 (↑KIR6.2 levels in plasma membrane, ↑GFAP+ cell density)	IHC (WB)	9, 12, 18-26mo	Alveus, SO, CA1 DG	[161]
GFAP colocalization with NOX2 (↑NOX2protein levels, ↑ signal intensity in GFAP+ cells)	IHC (WB)	18mo	HPC	[162]
GFAP colocalization with β1-integrin (↑β1-integrin protein levels, ↑ signal intensity in GFAP+ cells)	IHC (WB)	22-24mo	CA1 (not in0 CA3, DG, CTX	[162]
GFAP colocalization with RAGE (↑RAGE levels, ↑ GFAP+ cell density)	IHC (WB)	12mo	HPC	[25]
GFAP partial colocalization with NG2, astrocytes and oligodendrocyte progenitor cells cluster around the same Aβ plaques	IHC	24mo	HPC	[327]
GFAP colocalization with PHGDH (NC PHGDH protein levels)	IHC (WB, IHC)	6mo	HPC	[325]
GFAP colocalization with *Serpina3n* (RNA expression enriched in the soma and processes of Tau-associated astrocytes vs non-Tau-associated astrocytes)	IHC & *in situ* hybridization	21mo	CA1, SR	[328]
Observation: GFAP association with Tau granules	IHC (Tau5 IgG1 & T22 antibody)	21mo	CA1, SR	[328]

S100β colocalization with GRP78 (↑ protein levels & stained area)	IHC (WB)	12mo	HPC	[25]
Observation: Reactive astrocytes around plaque	IHC	15mo	HPC	[280]
Observation: Astrocytes were found in areas displaying glucose hypometabolism	IHC	11mo	EC, HPC	[329]
Observation: Hypertrophic astrocytes. Intense S100 staining of astrocyte processes, glia enwrapped Aβ	IHC	9, 18-24mo	Retina	[330]

Supplementary Table S3. Main astrocytic findings from LOAD and LOAD/FAD mouse models. All comparisons are versus age-matched wild-type mice unless otherwise mentioned. Abbreviations: ↑ = upregulation; ↓ = downregulation; NC = no significant change; ? = contradicting data * = missing data; BS = Brain Stem, CB = Cerebellum, CA = Cornu Ammonis, CST = Corticospinal Tract, CTX = Cortex, DG = Dentate Gyrus, ELISA = Enzyme-linked Immunosorbent Assay, EC = Entorhinal Cortex, FACS = Fluorescence-activated Cell Sorter, FtC = Frontal Cortex, GJ = Gap Junctions, HPC = Hippocampus, IHC = Immunohistochemistry, KI = Knock-in, LM = Laconosum Moleculare, mo = months old, ParC = Parietal Cortex, qPCR = Quantitative Polymerase Chain Reaction, RGCL = Retinal Ganglion Cell Layer, SC = Spinal Cord, SO = Stratum Oriens, SP = Stratum Pyramidale, SR = Stratum Radiatum, Tg = Transgenic mice, vs = versus (comparison), WB = Western Blot, WT = Wild Type. For additional synonyms for individual mouse models we recommend referring to www.informatics.jax.org. For space throughout the table the three separate lines APOE2, APOE3, APOE4 are written as APOE2/3/4.

Finding	Method	Age	Brain Area	References
APOE2/3/4-KI (targeted replacement) (also known as ApoE3-TR/ApoE4-TR)				
↓ *Abca1*, ↓ABCA1 and APOE in APOE4 vs APOE3 mice. ↑ARF6 in APOE4 vs APOE3 mice.	WB, PCR	4mo (male)	HPC	[331]
↑ spontaneous Ca^{2+} transient amplitude in APOE4 vs APOE3 male mice (NC in females)<br>NC spontaneous Ca^{2+} transient frequency in APOE4 vs APOE3 male mice (NC in females)	IHC (Ca^{2+} imaging – fluo4/AM)	9-12-week-old (males and females separately)	SR	[332]
↑ ATP-induced Ca^{2+} transient amplitude in APOE4 vs APOE3 male mice (NC in females)<br>NC ATP-induced Ca^{2+} transient frequency in APOE4 vs APOE3 male mice (NC in females)	IHC (fluo4/AM followed by ATP stimulation)	9-12-week-old (males and females separately)	SR	[332]
↑ post-ATP-induced Ca^{2+} transient amplitude in APOE4 vs APOE3 male mice (NC in females)<br>NC post-ATP-induced Ca^{2+} transient frequency in APOE4 vs APOE3 male mice (NC in females)	IHC (fluo4/AM followed by ATP stimulation)	9-12-week-old (males and females separately)	SR	[332]
GFAP does <u>not</u> colocalize with IgG	IHC	3, 10mo?	CTX, HPC	[333]
APOE2/3/4-KI (targeted replacement)				
NC GFAP+ cell density	IHC	4mo	HPC	[168]
NC IL-1β, IL-6, TNF-*α*	ELISA	4mo	HPC	[168]
↑ BrdU+/S100β+ cells 3 days & 4, 10 weeks post-injection in $Apoe^{-/-}$ vs WT, APOE-KI	IHC	6-7mo (female)	HPC	[334]
Tg(APOE2/3/4);$Apoe^{-/-}$				
↑ number of PAS+ clusters in astrocytes with age in $Apoe^{-/-}$<br>NC number of PAS+ clusters in astrocytes with age in APOE2, 3 or 4	Periodic acid Schiff (PAS) staining	4, 6, 8, 12mo	HPC	[335]
Observations: Similar GFAP protein levels & GFAP+ cell density in $Apoe^{-/-}$ & WT mice	WB, IHC	6mo (male)	HPC	[336]
Tg(Gfap-APOE3/4);$Apoe^{-/-}$				
↑ BrdU+/S100β+ cells 4 weeks post-injection in $Apoe^{-/-}$, Gfap-APOE3 & Gfap-APOE4 vs WT	IHC	6-7mo (female)	HPC	[334]
No fragmentation of APOE in Gfap-APOE3/4;$Apoe^{-/-}$ mice<br>Fragmentation of APOE in humans & Nse-APOE3/4;$Apoe^{-/-}$ mice	WB	8-10mo	Total	[337]

Tg(GFAP-APOE3/4;Apoe$^{-/-}$				
NC GFAP	WB	14-15mo	CTX, HPC	[338]
Tg(Thy1-APOE4) v Tg(hPDGF-APOE4) v Tg(hGFAP-APOE4) v Tg(PGK-APOE4)				
Observation: ↑ GFAP+ cells Thy1- & PDGF-APOE4 vs GFAP-, PGK-APOE4 and WT	IHC	8, 18mo	ParC, HPC, CST, Ventral Horn	[81,339]
APOE*R61T				
NC GFAP signal intensity	IHC	12mo (male)	CA1, CA3	[340]
↓ GLT1 signal intensity	IHC	12mo (male)	HPC	[340]
↓ ApoE signal intensity	IHC, WB	12mo (male)	HPC	[340]
PSEN1de9;Apoe$^{-/-}$ vs PSEN1de9;GFAP-ApoE$^{-/-}$				
↑ GFAP and *Gfap* in APP/PSEN1 vs WT, partially rescued in Apoe$^{-/-}$;APP/PSEN1 & Gfap-Apoe$^{-/-}$;APP/PSEN1 mice	WB, qPCR	12mo	CTX, HPC & Total	[170]
↑ GFAP+ cell density & cells per plaque in APP/PSEN1, partially rescued in the two Apoe$^{-/-}$	IHC	12mo	CTX, HPC & Total	[170]
↑ APOE in APP/PSEN1 from 3mo	WB	1, 3, 6, 12mo (male)	Total	[170]
↓ *Apoe* in CTX of Apoe$^{-/-}$;APPswe/PSEN1dE9 and Gfap-Apoe$^{-/-}$;APPswe/PSEN1dE9 vs WT<br>↓ *ApoE* in HPC of Apoe$^{-/-}$;APP/PSEN1 vs APP/PSEN1 and WT ↓ *ApoE* in hippocampus of Gfap-Apoe$^{-/-}$;APP/PSEN1 vs APP/PSEN1	qPCR	12mo	CTX, HPC	[170]
↑ *STAT3* & TGF-β<br>↑ pStat3/Stat3, pSmad2/Smad2 in APP/PSEN1, partially rescued in Apoe$^{-/-}$;APPswe/PSEN1dE9 and Gfap-Apoe$^{-/-}$;APPswe/PSEN1dE9	WB, qPCR	12mo	Total	[170]
↑ S100β in APPswe/PSEN1dE9, Apoe$^{-/-}$;APPswe/PSEN1dE9 and Gfap-Apoe$^{-/-}$;APPswe/PSEN1dE9 vs WT<br>NC *S100β* in APPswe/PSEN1dE9, Apoe$^{-/-}$;APPswe/PSEN1dE9 and Gfap-Apoe$^{-/-}$;APPswe/PSEN1dE9 vs WT	WB, qPCR	12mo	Total	[170]
NC Smad2, Hevin, SPARC	WB	12mo	Total	[170]
Tg(GFAP-Apoe3/4)xTg(APPSw/PSEN1de9)85 (also known as Apoe3/AD and Apoe4/AD)				
↑ IFNγ, MCP-1, MIP-1a, SCF, Rantes, b-NGF (cytokines)	ELISA	6mo	Total	[173]
Apoe$^{-/-}$;Tg(APP*V717F) vs Apoe$^{+/-}$;Tg(APP*V717F) vs Apoe$^{+/+}$;Tg(APP*V717F)				
Observation: Reduced GFAP signal intensity	IHC	21-22mo	CTX, HPC	[171]
Tg(Thy1-APOE4/APP*V717F/Thy1-PSEN1*A246E) vs Tg(Gfap-APOE4/APP*V717F/Thy1-PSEN1*A246E)				
GFAP colocalization with APOE in Thy1-APOE4;APP/PSEN1<br>GFAP did <u>not</u> colocalize with APOE in Thy1-APOE4;APP/PSEN1	IHC	15mo (female)	Perivascular areas	[175]
APOE4-KI;Apoe$^{-/-}$;Tg(PRNP-APPSweInd)8 (APOE4-KI is a targeted replacement) (also known as TR-APOE4;Apoe-/-;CRND8)				
↑ GFAP in APOE4 / CRND8, ↑ further in CRND8	WB	4, 7mo?	Total	[341]
↑ *Il1β* in CRND8 vs WT	qPCR	4, 7mo?	Total	[341]
NC *Tnfα* in CRND8 vs WT	qPCR	4, 7mo?	Total	[341]

GFAP negatively correlates with circadian rhythm measurements, nest construction, and positively correlates with IL-1β	Correlation analysis	4, 7mo?	Total	[341]
APOE2/3/4-KI;Tg(APPSwFlLon/PSEN1*M146L*L286V) (also known as APOE2/3/4-KI;5xFAD)				
↑ IL-1β	ELISA	6mo	CTX	[172]
Observation: Prominent GFAP signal intensity in subiculum and deep layers of the cortex in all three APOE2/3/4-KI / 5xFAD	IHC	6mo	CTX, HPC	[172]
APOE2/3/4-KI;Tg(Prnp-MAPT*P301S) (also known as P301S$^{hE/hE,}$ TE2/3/4) vs Tg(Prnp-MAPT*P301S);Apoe$^{-/-}$ (also known as TEKO)				
↑ GFAP in Tau APOE4-KI	WB	9mo	CTX	[176]
↑ A1, pan-reactive genes in TE4 9mo vs TEKO 9mo & TE4 4mo (no upregulation in hApoE3, hApoE4 mice vs WT 9mo)	Microfluidics qPCR	3, 9mo	CTX	[176]
Correlation between ↑ hippocampal / piriform GFAP area and ↓ hippocampal / piriform volume, respectively	IHC	9mo	CTX, HPC	[176]
Trem2$^{-/-}$;Tg(Prnp-MAPT*P301S) (also known as T2$^{-/-}$PS)				
↓ *Gfap, ApoE, Il1α, Il1β, Tnfα, C1q* in Trem2$^{-/-}$;PS19 vs PS19	qPCR	9mo	CTX	[179]
↓ GFAP+ area in TREM2$^{-/-}$;PS19 vs PS19	IHC	9mo	Piriform CTX, HPC	[179]
↓ GFAP/Iba1 area correlation	IHC	9mo	Piriform CTX, HPC	[179]
Trem2$^{-/-}$;Tg(APPSwFlLon/PSEN1*M146L*L286V) (also known as Trem2$^{-/-}$ 5xFAD) vs Trem2*R47H;Tg(APPSwFlLon/PSEN1*M146L*L286V) (also known as TREM2-R47H 5XFAD)				
Observation: ↓ Astrocytes (identified using the markers: *Slc1a2*, *Gja1*, *Aqp4*) seen in 5xFAD compared to WT, Trem2$^{-/-}$, Trem2$^{-/-}$;5xFAD<br>Observation: ↑ Astrocytes (identified using the markers: *GFAP*, *AQP4)* in human AD and AD with TREM2^{R62H} patients compared to control	snRNA-seq from bulk tissue	7mo	Total	[36]

Supplemental References

222. Yi, C.; Mei, X.; Ezan, P.; Mato, S.; Matias, I.; Giaume, C.; Koulakoff, A. Astroglial connexin43 contributes to neuronal suffering in a mouse model of Alzheimer's disease. *Cell Death Differ.* **2016**, *23*, 1691–1701, doi:10.1038/cdd.2016.63.

223. Orre, M.; Kamphuis, W.; Osborn, L.M.; Jansen, A.H.P.; Kooijman, L.; Bossers, K.; Hol, E.M. Isolation of glia from Alzheimer's mice reveals inflammation and dysfunction. *Neurobiol. Aging* **2014**, *35*, 2746–2760, doi:10.1016/j.neurobiolaging.2014.06.004.

224. Pan, J.; Ma, N.; Yu, B.; Zhang, W.; Wan, J. Transcriptomic profiling of microglia and astrocytes throughout aging. *J. Neuroinflammation* **2020**, *17*, 1–19, doi:10.1186/s12974-020-01774-9.

225. Kuchibhotla, K. V.; Lattarulo, C.R.; Hyman, B.T.; Bacskai, B.J. Synchronous Hyperactivity and Intercellular Calcium Waves in Astrocytes in Alzheimer Mice. *Science (80-.).* **2009**, *323*, 1211–1215, doi:10.1126/science.1169096.

226. Lee, K.I.; Lee, H. Te; Lin, H.C.; Tsay, H.J.; Tsai, F.C.; Shyue, S.K.; Lee, T.S. Role of transient receptor potential ankyrin 1 channels in Alzheimer's disease. *J. Neuroinflammation* **2016**, *13*, 1–16, doi:10.1186/s12974-016-0557-z.

227. Shrivastava, A.N.; Kowalewski, J.M.; Renner, M.; Bousset, L.; Koulakoff, A.; Melki, R.; Giaume, C.; Triller, A. β-amyloid and ATP-induced diffusional trapping of astrocyte and neuronal metabotropic glutamate type-5 receptors. *Glia* **2013**, *61*, 1673–1686, doi:10.1002/glia.22548.

228. Lv, J.; Ma, S.; Zhang, X.; Zheng, L.; Ma, Y.; Zhao, X.; Lai, W.; Shen, H.; Wang, Q.; Ji, J. Quantitative proteomics reveals that PEA15 regulates astroglial Aβ phagocytosis in an Alzheimer's disease mouse model. *J. Proteomics* **2014**, *110*, 45–58, doi:10.1016/j.jprot.2014.07.028.

229. Kim, S.; Lee, D.; Song, J.C.; Cho, S.J.; Yun, S.M.; Koh, Y.H.; Song, J.; Johnson, G.V.W.; Jo, C. NDP52 associates with phosphorylated tau in brains of an Alzheimer disease mouse model. *Biochem. Biophys. Res. Commun.* **2014**, *454*, 196–201, doi:10.1016/j.bbrc.2014.10.066.

230. Plá, V.; Paco, S.; Ghezali, G.; Ciria, V.; Pozas, E.; Ferrer, I.; Aguado, F. Secretory sorting receptors carboxypeptidase e and secretogranin III in amyloid β-associated neural degeneration in alzheimer's disease. *Brain Pathol.* **2013**, *23*, 274–284, doi:10.1111/j.1750-3639.2012.00644.x.

231. Kniewallner, K.M.; de Sousa, D.M.B.; Unger, M.S.; Mrowetz, H.; Aigner, L. Platelets in Amyloidogenic Mice Are Activated and Invade the Brain. *Front. Neurosci.* **2020**, *14*, doi:10.3389/fnins.2020.00129.

232. Ma, W.; Wu, M.; Zhou, S.; Tao, Y.; Xie, Z.; Zhong, Y. Reduced Smoothened level rescues Aβ-induced memory deficits and neuronal inflammation in animal models of Alzheimer's disease. *J. Genet. Genomics* **2018**, *45*, 237–246, doi:10.1016/j.jgg.2018.05.001.

233. Slowik, A.; Merres, J.; Elfgen, A.; Jansen, S.; Mohr, F.; Wruck, C.J.; Pufe, T.; Brandenburg, L.O. Involvement of formyl peptide receptors in receptor for advanced glycation end products (RAGE) - And amyloid beta 1-42-induced signal transduction in glial cells. *Mol. Neurodegener.* **2012**, *7*, 1–18, doi:10.1186/1750-1326-7-55.

234. Papadinitriou, C.; Celikkaya, H.; Cosacak, M.I.; Mashkaryan, V.; Bray, L.; Bhattara, P.; Brand, K.; Al., E. 3D Culture Method for Alzheimer's Disease Modeling Reveals Interleukin-4 RescuesAb42-Induced Loss of Human Neural Stem Cell Plasticity. *Dev. Cell* **2018**, *46*, 85–101.

235. Xing, S.; Shen, D.; Chen, C.; Wang, J.; Yu, Z. Early induction of oxidative stress in a mouse model of Alzheimer's disease with heme oxygenase activity. *Mol. Med. Rep.* **2014**, *10*, 599–604, doi:10.3892/mmr.2014.2252.

236. Orr, A.G.; Hsiao, E.C.; Wang, M.M.; Ho, K.; Kim, D.H.; Wang, X.; Guo, W.; Kang, J.; Yu, G.Q.; Adame, A.; et al. Astrocytic adenosine receptor A2A and Gs-coupled signaling regulate memory. *Nat. Neurosci.* **2015**, *18*, 423–439, doi:10.1038/nn.3930.

237. Liu, L.; Lai, Y.J.; Zhao, L.G.; Chen, G.J. Increased expression of Myc-interacting zinc finger protein 1 in APP/PS1 mice. *Exp. Ther. Med.* **2017**, *14*, 5751–5756, doi:10.3892/etm.2017.5289.

238. Ko, C.-Y.; Wang, W.-L.; Wang, S.-M.; Chu, Y.-Y.; Chang, W.-C.; Wang, J.-M. Glycogen synthase kinase-3bemediated CCAAT/enhancer-binding protein delta phosphorylation in astrocytes promot es migration and activation of microglia/ macrophages. *Neurobiol. Aging* **2014**, *35*, 24–34, doi:10.1016/j.neurobiolaging.2013.07.021.

239. Lin, S.; Wei, L.; Ping, Y.; Xia, L.; Xiao, S. Upregulated BMP6 pathway involved in the pathogenesis of Aβ toxicity in vivo. *Neurosci. Lett.* **2018**, *664*, 152–159, doi:10.1016/j.neulet.2017.11.022.

240. Wang, L.; Xu, X. Bin; You, W.W.; Lin, X.X.; Li, C.T.; Qian, H.R.; Zhang, L.H.; Yang, Y. The cytoplasmic nuclear shuttling of Beclin 1 in neurons with Alzheimer's disease-like injury. *Neurosci. Lett.* **2017**, *661*, 63–70, doi:10.1016/j.neulet.2017.09.055.

241. Canepa, E.; Borghi, R.; Vina, J.; Traverso, N.; Gambini, J.; Domenicotti, C.; Marinari, U.M.; Poli, G.; Pronzato, M.A.; Ricciarelli, R. Cholesterol and Amyloid-β: Evidence for a Cross-Talk between Astrocytes and Neuronal Cells. *J. Alzheimer's Dis.* **2011**, *25*, 645–653, doi:10.3233/JAD-2011-110053.

242. Ostapchenko, V.G.; Beraldo, F.H.; Mohammad, A.H.; Xie, Y.F.; Hirata, P.H.F.; Magalhaes, A.C.; Lamour, G.; Li, H.; Maciejewski, A.; Belrose, J.C.; et al. The prion protein ligand, stress-inducible phosphoprotein 1, regulates amyloid-β oligomer toxicity. *J. Neurosci.* **2013**, *33*, 16552–16564, doi:10.1523/JNEUROSCI.3214-13.2013.

243. Ahn, K.C.; Learman, C.R.; Dunbar, G.L.; Maiti, P.; Jang, W.C.; Cha, H.C.; Song, M.S. Characterization of Impaired Cerebrovascular Structure in APP/PS1 Mouse Brains. *Neuroscience* **2018**, *385*, 246–254, doi:10.1016/j.neuroscience.2018.05.002.

244. Giannoni, P.; Arango-Lievano, M.; Neves, I. Das; Rousset, M.C.; Baranger, K.; Rivera, S.; Jeanneteau, F.; Claeysen, S.; Marchi, N. Cerebrovascular pathology during the progression of experimental Alzheimer's disease. *Neurobiol. Dis.* **2016**, *88*, 107–117, doi:10.1016/j.nbd.2016.01.001.

245. Sompol, P.; Furman, J.L.; Pleiss, M.M.; Kraner, S.D.; Artiushin, I.A.; Batten, S.R.; Quintero, J.E.; Simmerman, L.A.; Beckett, T.L.; Lovell, M.A.; et al. Calcineurin/NFAT signaling in activated astrocytes drives network hyperexcitability in Aβ-bearing mice. *J. Neurosci.* **2017**, *37*, 6132–6148, doi:10.1523/JNEUROSCI.0877-17.2017.

246. Iram, T.; Trudler, D.; Kain, D.; Kanner, S.; Galron, R.; Vassar, R.; Barzilai, A.; Blinder, P.; Fishelson, Z.; Frenkel, D. Astrocytes from old Alzheimer's disease mice are impaired in Aβ uptake and in neuroprotection. *Neurobiol. Dis.* **2016**, *96*, 84–94, doi:10.1016/j.nbd.2016.08.001.

247. Py, N.A.; Bonnet, A.E.; Bernard, A.; Marchalant, Y.; Charrat, E.; Checler, F.; Khrestchatisky, M.; Baranger, K.; Rivera, S. Differential spatio-temporal regulation of MMPs in the 5xFAD mouse model of Alzheimer's disease: Evidence for a pro-amyloidogenic role of MT1-MMP. *Front. Aging Neurosci.* **2014**, *6*, 1–17, doi:10.3389/fnagi.2014.00247.

248. Ye, B.; Shen, H.; Zhang, J.; Zhu, Y.G.; Ransom, B.R.; Chen, X.C.; Ye, Z.C. Dual pathways mediate β-amyloid stimulated glutathione release from astrocytes. *Glia* **2015**, *63*, 2208–2219, doi:10.1002/glia.22886.

249. Son, S.M.; Nam, D.W.; Cha, M.Y.; Kim, K.H.; Byun, J.; Ryu, H.; Mook-Jung, I. Thrombospondin-1 prevents amyloid beta-mediated synaptic pathology in Alzheimer's disease. *Neurobiol. Aging* **2015**, *36*, 3214–3227, doi:10.1016/j.neurobiolaging.2015.09.005.

250. Li, W.; Poteet, E.; Xie, L.; Liu, R.; Wen, Y.; Yang, S.-H. Regulation of matrix metalloproteinase 2 by oligomeric amyloid β protein. *Brain Res.* **2011**, *1387*, 141–148, doi:10.1016/j.brainres.2011.02.078.

251. Maruyama, T.; Wada, H.; Abe, Y.; Niikura, T. Alteration of global protein SUMOylation in neurons and astrocytes in response to Alzheimer's disease-associated insults. *Biochem. Biophys. Res. Commun.* **2018**, *500*, 470–475, doi:10.1016/j.bbrc.2018.04.104.

252. Wei, Z.; Chen, X.-C.; Song, Y.; Pan, X.-D.; Dai, X.-M.; Zhang, J.; Cui, X.-L.; Wu, X.-L.; Zhu, Y.-G. Amyloid β Protein Aggravates Neuronal Senescence and Cognitive Deficits in 5XFAD Mouse Model of Alzheimer's Disease. *Chin. Med. J. (Engl).* **2016**, *129*, 1835–1844, doi:10.4103/0366-6999.186646.

253. Gaudel, F.; Stephan, D.; Landel, V.; Sicard, G.; Féron, F.; Guiraudie-Capraz, G. Expression of the Cerebral Olfactory Receptors Olfr110/111 and Olfr544 Is Altered During Aging and in Alzheimer's Disease-Like Mice. *Mol. Neurobiol.* **2019**, *56*, 2057–2072, doi:10.1007/s12035-018-1196-4.

254. Liang, Y.; Raven, F.; Ward, J.F.; Zhen, S.; Zhang, S.; Sun, H.; Miller, S.J.; Choi, S.H.; Tanzi, R.E.; Zhang, C. Upregulation of Alzheimer's Disease Amyloid-β Protein Precursor in Astrocytes Both in vitro and in vivo. *J. Alzheimer's Dis.* **2020**, 1–12, doi:10.3233/JAD-200128.

255. Mirzaei, N.; Tang, S.P.; Ashworth, S.; Coello, C.; Plisson, C.; Passchier, J.; Selvaraj, V.; Tyacke, R.J.; Nutt, D.J.; Sastre, M. In vivo imaging of microglial activation by positron emission tomography with [11C]PBR28 in the 5XFAD model of Alzheimer's disease. *Glia* **2016**, *64*, 993–1006, doi:10.1002/glia.22978.

256. Shen, H.; Pan, X.D.; Zhang, J.; Zeng, Y.Q.; Zhou, M.; Yang, L.M.; Ye, B.; Dai, X.M.; Zhu, Y.G.; Chen, X.C. Endoplasmic reticulum stress induces the early appearance of pro-apoptotic and anti-apoptotic proteins in neurons of five familial alzheimer's disease mice. *Chin. Med. J. (Engl).* **2016**, *129*, 2845–2852, doi:10.4103/0366-6999.194643.

257. Boscia, F.; Pannaccione, A.; Ciccone, R.; Casamassa, A.; Franco, C.; Piccialli, I.; de Rosa, V.; Vinciguerra, A.; Di Renzo, G.; Annunziato, L. The expression and activity of KV3.4 channel subunits are precociously upregulated in astrocytes exposed to Aβ oligomers and in astrocytes of Alzheimer's disease Tg2576 mice. *Neurobiol. Aging* **2017**, *54*, 187–198, doi:10.1016/j.neurobiolaging.2017.03.008.

258. Rodriguez-Vieitez, E.; Ni, R.; Gulyás, B.; Tóth, M.; Häggkvist, J.; Halldin, C.; Voytenko, L.; Marutle, A.; Nordberg, A. Astrocytosis precedes amyloid plaque deposition in Alzheimer APPswe transgenic mouse brain: a correlative positron emission tomography and in vitro imaging study. *Eur. J. Nucl. Med. Mol. Imaging* **2015**, *42*, 1119–1132, doi:10.1007/s00259-015-3047-0.

259. Tratnjek, L.; Živin, M.; Glavan, G. Up-regulation of synaptotagmin IV within amyloid plaqueassociated dystrophic neurons in Tg2576 mouse model of alzheimer's disease. *Croat. Med. J.* **2013**, *54*, 419–428, doi:10.3325/cmj.2013.54.419.

260. Yin, K.-J.; Cirrito, J.R.; Yan, P.; Hu, X.; Xiao, Q.; Pan, X.; Bateman, R.; Al., E. Matrix Metalloproteinases Expressed by Astrocytes Mediate Extracellular Amyloid-®Peptide Catabolism. *J. Neurosci.* **2006**, *26*, 10939–10948, doi:10.1523/JNEUROSCI.2085-06.2006.

261. Carrasco, J.; Adlard, P.; Cotman, C.; Quintana, A.; Penkowa, M.; Xu, F.; Van Nostrand, W.E.; Hidalgo, J. Metallothionein-I and -III expression in animal models of Alzheimer disease. *Neuroscience* **2006**, *143*, 911–922, doi:10.1016/j.neuroscience.2006.08.054.

262. Raha, A.A.; Vaishnav, R.A.; Friedland, R.P.; Bomford, A.; Raha-Chowdhury, R. The systemic iron-regulatory proteins hepcidin and ferroportin are reduced in the brain in Alzheimer's disease. *Acta Neuropathol. Commun.* **2014**, *2*, 1–19, doi:10.1186/2051-5960-1-55.

263. George, A.J.; Holsinger, R.M.D.; McLean, C.A.; Tan, S.S.; Scott, H.S.; Cardamone, T.; Cappai, R.; Masters, C.L.; Li, Q.X. Decreased phosphatidylethanolamine binding protein expression correlates with Aβ accumulation in the Tg2576 mouse model of Alzheimer's disease. *Neurobiol. Aging* **2006**, *27*, 614–623, doi:10.1016/j.neurobiolaging.2005.03.014.

264. Maioli, S.; Lodeiro, M.; Merino-Serrais, P.; Falahati, F.; Khan, W.; Puerta, E.; Codita, A.; Rimondini, R.; Ramirez, M.J.; Simmons, A.; et al. Alterations in brain leptin signalling in spite of unchanged CSF leptin levels in Alzheimer's disease. *Aging Cell* **2015**, *14*, 122–129, doi:10.1111/acel.12281.

265. Rodrigo, J.; Fernández-Vizarra, P.; Castro-Blanco, S.; Bentura, M.L.; Nieto, M.; Gómez-Isla, T.; Martínez-Murillo, R.; Martínez, A.; Serrano, J.; Fernández, A.P. Nitric oxide in the cerebral cortex of amyloid-precursor protein (SW) Tg2576 transgenic mice. *Neuroscience* **2004**, *128*, 73–89, doi:10.1016/j.neuroscience.2004.06.030.

266. Huesa, G.; Baltrons, M.A.; Gómez-Ramos, P.; Morán, A.; García, A.; Hidalgo, J.; Francés, S.; Santpere, G.; Ferrer, I.; Galea, E. Altered distribution of RhoA in Alzheimer's disease and AβPP overexpressing mice. *J. Alzheimer's Dis.* **2010**, *19*, 37–56, doi:10.3233/JAD-2010-1203.

267. Terai, K.; Iwai, A.; Kawabata, S.; Sasamata, M.; Miyata, K.; Yamaguchi, T. Apolipoprotein E deposition and astrogliosis are associated with maturation of β-amyloid plaques in βAPPswe transgenic mouse: Implications for the pathogenesis of Alzheimer's disease. *Brain Res.* **2001**, *900*, 48–56, doi:10.1016/S0006-8993(01)02202-8.

268. Motoi, Y.; Itaya, M.; Mori, H.; Mizuno, Y.; Iwasaki, T.; Hattori, H.; Haga, S.; Ikeda, K. Apolipoprotein E receptor 2 is involved in neuritic plaque formation in APP sw mice. *Neurosci. Lett.* **2004**, *368*, 144–147, doi:10.1016/j.neulet.2004.06.081.

269. Abbas, N.; Bednar, I.; Mix, E.; Marie, S.; Paterson, D.; Ljungberg, A.; Morris, C.; Winblad, B.; Nordberg, A.; Zhu, J. Up-regulation of the inflammatory cytokines IFN-γ and IL-12 and down-regulation of IL-4 in cerebral cortex regions of APPSWE transgenic mice. *J. Neuroimmunol.* **2002**, *126*, 50–57, doi:10.1016/S0165-5728(02)00050-4.

270. Hartlage-Rübsamen, M.; Zeitschel, U.; Apelt, J.; Gärtner, U.; Franke, H.; Stahl, T.; Günther, A.; Schliebs, R.; Penkowa, M.; Bigl, V.; et al. Astrocytic expression of the Alzheimer's disease β-secretase (BACE1) is stimulus-dependent. *Glia* **2003**, *41*, 169–179, doi:10.1002/glia.10178.

271. Lee, J.Y.; Kweon, H.S.; Cho, E.; Lee, J.Y.; Byun, H.R.; Kim, D.H.; Kim, Y.H.; Han, P.L.; Koh, J.Y. Upregulation of tPA/plasminogen proteolytic system in the periphery of amyloid deposits in the Tg2576 mouse model of Alzheimer's disease. *Neurosci. Lett.* **2007**, *423*, 82–87, doi:10.1016/j.neulet.2007.06.037.

272. Nishiyama, K.; Trapp, B.D.; Ikezu, T.; Ransohoff, R.M.; Tomita, T.; Iwatsubo, T.; Kanazawa, I.; Hsiao, K.K.; Lisanti, M.P.; Okamoto, T. Caveolin-3 upregulation activates β-secretase-mediated cleavage of the amyloid precursor protein in Alzheimer's disease. *J. Neurosci.* **1999**, *19*, 6538–6548, doi:10.1523/jneurosci.19-15-06538.1999.

273. Apelt, J.; Ach, K.; Schliebs, R. Aging-related down-regulation of neprilysin, a putative β-amyloid-degrading enzyme, in transgenic Tg2576 Alzheimer-like mouse brain is accompanied by an astroglial upregulation in the vicinity of β-amyloid plaques. *Neurosci. Lett.* **2003**, *339*, 183–186, doi:10.1016/S0304-3940(03)00030-2.

274. Steinhoff, T.; Moritz, E.; Wollmer, M.A.; Mohajeri, M.H.; Kins, S.; Nitsch, R.M. Increased cystatin C in astrocytes of transgenic mice expressing the K670N-M671L mutation of the amyloid precursor protein and deposition in brain amyloid plaques. *Neurobiol. Dis.* **2001**, *8*, 647–654, doi:10.1006/nbdi.2001.0412.

275. Hartlage-Rübsamen, M.; Ratz, V.; Zeitschel, U.; Finzel, L.; Machner, L.; Köppen, J.; Schulze, A.; Demuth, H.U.; von Hörsten, S.; Höfling, C.; et al. Endogenous mouse huntingtin is highly abundant in cranial nerve nuclei, co-aggregates to Abeta plaques and is induced in reactive astrocytes in a transgenic mouse model of Alzheimer's disease. *Acta Neuropathol. Commun.* **2019**, *7*, 79, doi:10.1186/s40478-019-0726-2.

276. Apelt, J.; Schliebs, R. β-Amyloid-induced glial expression of both pro- and anti-inflammatory cytokines in cerebral cortex of aged transgenicTg2576 mice with Alzheimer plaque pathology. *Brain Res.* **2001**, *894*, 21–30, doi:10.1016/s0006-8993(00)03176-0.

277. Pugazhenthi, S.; Wang, M.; Pham, S.; Sze, C.I.; Eckman, C.B. Downregulation of CREB expression in Alzheimer's brain and in Aβ-treated rat hippocampal neurons. *Mol. Neurodegener.* **2011**, *6*, 1–16, doi:10.1186/1750-1326-6-60.

278. Ameen-Ali, K.E.; Simpson, J.E.; Wharton, S.B.; Heath, P.R.; Sharp, P.S.; Brezzo, G.; Berwick, J. The Time Course of Recognition Memory Impairment and Glial Pathology in the hAPP-J20 Mouse Model of Alzheimer's Disease. *J. Alzheimer's Dis.* **2019**, *68*, 609–624, doi:10.3233/JAD-181238.

279. Fu, Y.H.; Rusznák, Z.; Kwok, J.B.J.; Kim, W.S.; Paxinos, G. Age-dependent alterations of the hippocampal cell composition and proliferative potential in the hAβPPSwInd-J20 mouse. *J. Alzheimer's Dis.* **2014**, *41*, 1177–1192, doi:10.3233/JAD-132717.

280. Li, N.; Hu, P.; Xu, T.; Chen, H.; Chen, X.; Hu, J.; Yang, X.; Shi, L.; Luo, J.; Xu, J. iTRAQ-based Proteomic Analysis of APPSw,Ind Mice Provides Insights into the Early Changes in Alzheimer's Disease. *Curr. Alzheimer Res.* **2017**, *14*, 1109–1122, doi:10.2174/1567205014666170719165745.

281. Pomilio, C.; Pavia, P.; Gorojod, R.M.; Vinuesa, A.; Alaimo, A.; Galvan, V.; Kotler, M.L.; Beauquis, J.; Saravia, F. Glial alterations from early to late stages in a model of Alzheimer's disease: Evidence of autophagy involvement in Aβ internalization. *Hippocampus* **2016**, *26*, 194–210, doi:10.1002/hipo.22503.

282. Durand, D.; Carniglia, L.; Beauquis, J.; Caruso, C.; Saravia, F.; Lasaga, M. Astroglial mGlu3 receptors promote alpha-secretase-mediated amyloid precursor protein cleavage. *Neuropharmacology* **2014**, *79*, 180–189, doi:10.1016/j.neuropharm.2013.11.015.

283. Terwel, D.; Steffensen, K.R.; Verghese, P.B.; Kummer, M.P.; Gustafsson, J.Å.; Holtzman, D.M.; Heneka, M.T. Critical role of astroglial apolipoprotein E and liver X receptor-α expression for microglial Aβ phagocytosis. *J. Neurosci.* **2011**, *31*, 7049–7059, doi:10.1523/JNEUROSCI.6546-10.2011.

284. Hagmeyer, S.; Romão, M.A.; Cristóvão, J.S.; Vilella, A.; Zoli, M.; Gomes, C.M.; Grabrucker, A.M. Distribution and relative abundance of S100 proteins in the brain of the APP23 Alzheimer's disease model mice. *Front. Neurosci.* **2019**, *13*, 1–10, doi:10.3389/fnins.2019.00640.

285. Wilhelmus, M.M.M.; De Jager, M.; Smit, A.B.; Van Der Loo, R.J.; Drukarch, B. Catalytically active tissue transglutaminase colocalises with Aβ pathology in Alzheimer's disease mouse models. *Sci. Rep.* **2016**, *6*, 1–12, doi:10.1038/srep20569.

286. Ji, B.; Maeda, J.; Sawada, M.; Ono, M.; Okauchi, T.; Inaji, M.; Zhang, M.R.; Suzuki, K.; Ando, K.; Staufenbiel, M.; et al. Imaging of peripheral benzodiazepine receptor expression as biomarkers of detrimental versus beneficial glial responses in mouse models of Alzheimer's and other CNS pathologies. *J. Neurosci.* **2008**, *28*, 12255–12267, doi:10.1523/JNEUROSCI.2312-08.2008.

287. Burbach, G.J.; Hellweg, R.; Haas, C.A.; Del Turco, D.; Deicke, U.; Abramowski, D.; Jucker, M.; Staufenbiel, M.; Deller, T. Induction of Brain-Derived Neurotrophic Factor in Plaque-Associated Glial Cells of Aged APP23 Transgenic Mice. *J. Neurosci.* **2004**, *24*, 2421–2430, doi:10.1523/JNEUROSCI.5599-03.2004.

288. Gärtner, U.; Brückner, M.K.; Krug, S.; Schmetsdorf, S.; Staufenbiel, M.; Arendt, T. Amyloid deposition in APP23 mice is associated with the expression of cyclins in astrocytes but not in neurons. *Acta Neuropathol.* **2003**, *106*, 535–544, doi:10.1007/s00401-003-0760-8.

289. Lüth, H.J.; Holzer, M.; Gärtner, U.; Staufenbiel, M.; Arendt, T. Expression of endothelial and inducible NOS-isoforms is increased in Alzheimer's disease, in APP23 transgenic mice and after experimental brain lesion in rat: Evidence for an induction by amyloid pathology. *Brain Res.* **2001**, *913*, 57–67, doi:10.1016/S0006-8993(01)02758-5.

290. Olsen, M.; Aguilar, X.; Sehlin, D.; Fang, X.T.; Antoni, G.; Erlandsson, A.; Syvänen, S. Astroglial Responses to Amyloid-Beta Progression in a Mouse Model of Alzheimer's Disease. *Mol. Imaging Biol.* **2018**, *20*, 605–614, doi:10.1007/s11307-017-1153-z.

291. Thomason, L.A.M.; Smithson, L.J.; Hazrati, L.N.; McLaurin, J.; Kawaja, M.D. Reactive astrocytes associated with plaques in TgCRND8 mouse brain and in human alzheimer brain express phosphoprotein enriched in astrocytes (PEA-15). *FEBS Lett.* **2013**, *587*, 2448–2454, doi:10.1016/j.febslet.2013.06.015.

292. Bouvier, D.S.; Jones, E. V.; Quesseveur, G.; Davoli, M.A.; Ferreira, T.A.; Quirion, R.; Mechawar, N.; Murai, K.K. High Resolution Dissection of Reactive Glial Nets in Alzheimer's Disease. *Sci. Rep.* **2016**, *6*, 1–15, doi:10.1038/srep24544.

293. Giovannini, M.G.; Cerbai, F.; Bellucci, A.; Melani, C.; Grossi, C.; Bartolozzi, C.; Nosi, D.; Casamenti, F. Differential activation of mitogen-activated protein kinase signalling pathways in the hippocampus of CRND8 transgenic mouse, a model of Alzheimer's disease. *Neuroscience* **2008**, *153*, 618–633, doi:10.1016/j.neuroscience.2008.02.061.

294. Dudal, S.; Krzywkowski, P.; Paquette, J.; Morissete, C.; Lacombe, D.; Tremblay, P.; Gervais, F. Inflammation occurs early during the Aβ deposition process in TgCRND8 mice. *Neurobiol. Aging* **2004**, *25*, 861–871, doi:10.1016/j.neurobiolaging.2003.08.008.

295. Palareti, G.; Legnani, C.; Cosmi, B.; Antonucci, E.; Erba, N.; Poli, D.; Testa, S.; Tosetto, A. Comparison between different D-Dimer cutoff values to assess the individual risk of recurrent venous thromboembolism: Analysis of results obtained in the DULCIS study. *Int. J. Lab. Hematol.* **2016**, *38*, 42–49, doi:10.1111/ijlh.12426.

296. Xu, F.; Grande, A.M.; Robinson, J.K.; Previti, M.L.; Vasek, M.; Davis, J.; Van Nostrand, W.E. Early-onset subicular microvascular amyloid and neuroinflammation correlate with behavioral deficits in vasculotropic mutant amyloid β-protein precursor transgenic mice. *Neuroscience* **2007**, *146*, 98–107, doi:10.1016/j.neuroscience.2007.01.043.

297. Fan, R.; DeFilippis, K.; Van Nostrand, W.E. Induction of complement proteins in a mouse model for cerebral microvascular Aβ deposition. *J. Neuroinflammation* **2007**, *4*, 1–8, doi:10.1186/1742-2094-4-22.

298. Baglietto-Vargas, D.; Sanchez-Mejias, E.; Navarro, V.; Jimenez, S.; Trujillo-Estrada, L.; Al., E. Dual roles of Aβ in proliferative

processes in an amyloidogenic model of Alzheimer's disease. *Sci. Rep.* **2017**, *7*, doi:10.1038/s41598-017-10353-7.

299. Boom, A.; Pochet, R.; Authelet, M.; Pradier, L.; Borghgraef, P.; Van Leuven, F.; Heizmann, C.W.; Brion, J.P. Astrocytic calcium/zinc binding protein S100A6 over expression in Alzheimer's disease and in PS1/APP transgenic mice models. *Biochim. Biophys. Acta - Mol. Cell Res.* **2004**, *1742*, 161–168, doi:10.1016/j.bbamcr.2004.09.011.

300. Cruz, N.F.; Ball, K.K.; Dienel, G.A. Astrocytic gap junctional communication is reduced in amyloid-ß-treated cultured astrocytes, but not in alzheimer's disease transgenic mice. *ASN Neuro* **2010**, *2*, 201–213, doi:10.1042/AN20100017.

301. Heneka, M.T.; Sastre, M.; Dumitrescu-Ozimek, L.; Dewachter, I.; Walter, J.; Klockgether, T.; Van Leuven, F. Focal glial activation coincides with increased BACE1 activation and precedes amyloid plaque deposition in APP[V717I] transgenic mice. *J. Neuroinflammation* **2005**, *2*, 1–12, doi:10.1186/1742-2094-2-22.

302. Zhu, S.; Wang, J.; Zhang, Y.; He, J.; Kong, J.; Wang, J.F.; Li, X.M. The role of neuroinflammation and amyloid in cognitive impairment in an APP/PS1 transgenic mouse model of Alzheimer's disease. *CNS Neurosci. Ther.* **2017**, *23*, 310–320, doi:10.1111/cns.12677.

303. Gordon, M.N.; Holcomb, L.A.; Jantzen, P.T.; DiCarlo, G.; Wilcock, D.; Boyett, K.W.; Connor, K.; Melachrino, J.; O'Callaghan, J.P.; Morgan, D. Time course of the development of Alzheimer-like pathology in the doubly transgenic PS1+APP mouse. *Exp. Neurol.* **2002**, *173*, 183–195, doi:10.1006/exnr.2001.7754.

304. Nakamura, K.; Ohya, W.; Fukanoshi, H.; Sakaguchi, G.; Kato, A.; Al., E. Possible Role of Scavenger Receptor SRCL in the Clearance of Amyloid-b in Alzheimer's Disease. *J. Neurosci. Res.* **2006**, *84*, 874–890, doi:10.1002/jnr.20992.

305. Peters, O.; Schipke, C.G.; Philipps, A.; Haas, B.; Pannasch, U.; Wang, L.P.; Benedetti, B.; Kingston, A.E.; Kettenmann, H. Astrocyte function is modified by alzheimer's disease-like pathology in aged mice. *J. Alzheimer's Dis.* **2009**, *18*, 177–189, doi:10.3233/JAD-2009-1140.

306. Arélin, K.; Kinoshita, A.; Whelan, C.M.; Irizarry, M.C.; Rebeck, G.W.; Strickland, D.K.; Hyman, B.T. LRP and senile plaques in Alzheimer's disease: Colocalization with apolipoprotein E and with activated astrocytes. *Mol. Brain Res.* **2002**, *104*, 38–46, doi:10.1016/S0169-328X(02)00203-6.

307. Edwards, S.R.; Khan, N.; Coulson, E.J.; Smith, M.T. Comparative studies of glial fibrillary acidic protein and brain-derived neurotrophic factor expression in two transgenic mouse models of Alzheimer's disease. *Clin. Exp. Pharmacol. Physiol.* **2020**, doi:10.1111/1440-1681.13363.

308. Castillo, E.; Leon, J.; Mazzei, G.; Abolhassani, N.; Haruyama, N.; Saito, T.; Saido, T.; Hokama, M.; Iwaki, T.; Ohara, T.; et al. Comparative profiling of cortical gene expression in Alzheimer's disease patients and mouse models demonstrates a link between amyloidosis and neuroinflammation. *Sci. Rep.* **2017**, *7*, 1–16, doi:10.1038/s41598-017-17999-3.

309. Shi, A.; Petrache, A.L.; Shi, J.; Ali, A.B. Preserved Calretinin Interneurons in an App Model of Alzheimer's Disease Disrupt Hippocampal Inhibition via Upregulated P2Y1 Purinoreceptors. *Cereb. Cortex* **2020**, 1272–1290, doi:10.1093/cercor/bhz165.

310. Li, K.Y.; Gong, P.F.; Li, J.T.; Xu, N.J.; Qin, S. Morphological and molecular alterations of reactive astrocytes without proliferation in cerebral cortex of an APP/PS1 transgenic mouse model and Alzheimer's patients. *Glia* **2020**, 1–16, doi:10.1002/glia.23845.

311. Reichenbach, N.; Delekate, A.; Breithausen, B.; Keppler, K.; Poll, S.; Schulte, T.; Peter, J.; Plescher, M.; Hansen, J.N.; Blank, N.; et al. P2Y1 receptor blockade normalizes network dysfunction and cognition in an Alzheimer's disease model. *J. Exp. Med.* **2018**, *215*, 1649–1663, doi:10.1084/jem.20171487.

312. Radbruch, H.; Mothes, R.; Bremer, D.; Seifert, S.; Köhler, R.; Pohlan, J.; Ostendorf, L.; Günther, R.; Leben, R.; Stenzel, W.; et al. Analyzing nicotinamide adenine dinucleotide phosphate oxidase activation in aging and vascular amyloid pathology. *Front. Immunol.* **2017**, *8*, 1–12, doi:10.3389/fimmu.2017.00844.

313. Löffler, T.; Flunkert, S.; Havas, D.; Schweinzer, C.; Uger, M.; Windisch, M.; Steyrer, E.; Hutter-Paier, B. Neuroinflammation and related neuropathologies in APPSL mice: Further value of this in vivo model of Alzheimer's disease. *J. Neuroinflammation* **2014**, *11*, 1–13, doi:10.1186/1742-2094-11-84.

314. Willis, M.; Kaufmann, W.A.; Wietzorrek, G.; Hutter-Paier, B.; Moosmang, S.; Humpel, C.; Hofmann, F.; Windisch, M.; Knaus, H.G.; Marksteiner, J. L-Type calcium channel CaV 1.2 in transgenic mice overexpressing human AβPP751 with the London (V717I) and Swedish (K670M/N671L) Mutations. *J. Alzheimer's Dis.* **2010**, *20*, 1167–1180, doi:10.3233/JAD-2010-091117.

315. Daschil, N.; Obermair, G.J.; Flucher, B.E.; Stefanova, N.; Hutter-Paier, B.; Windisch, M.; Humpel, C.; Marksteiner, J. CaV1.2 calcium channel expression in reactive astrocytes is associated with the formation of amyloid-β plaques in an Alzheimer's disease mouse model. *J. Alzheimer's Dis.* **2013**, *37*, 439–451, doi:10.3233/JAD-130560.

316. Tomiyama, T.; Matsuyama, S.; Iso, H.; Umeda, T.; Takuma, H.; Ohnishi, K.; Al., E. A Mouse Model ofAmyloid β Oligomers: Their Contribution to Synaptic Alteration, Abnormal Tau Phosphorylation, Glial Activation, and Neuronal Loss In Vivo. *Neurobiol. Aging* **2010**, *30*, 4845–4856, doi:10.1523/JNEUROSCI.5825-09.2010.

317. Brawek, B.; Chesters, R.; Klement, D.; Müller, J.; Lerdkrai, C.; Hermes, M.; Garaschuk, O. A bell-shaped dependence between amyloidosis and GABA accumulation in astrocytes in a mouse model of Alzheimer's disease. *Neurobiol. Aging* **2018**, *61*, 187–197, doi:10.1016/j.neurobiolaging.2017.09.028.

318. Blair, L.J.; Frauen, H.D.; Zhang, B.; Nordhues, B.A.; Bijan, S.; Lin, Y.C.; Zamudio, F.; Hernandez, L.D.; Sabbagh, J.J.; Selenica, M.L.B.; et al. Tau depletion prevents progressive blood-brain barrier damage in a mouse model of tauopathy. *Acta Neuropathol. Commun.* **2015**, *3*, 8, doi:10.1186/s40478-015-0186-2.

319. Ludvigson, A.E.; Luebke, J.I.; Lewis, J.; Peters, A. Structural abnormalities in the cortex of the rTg4510 mouse model of tauopathy: A light and electron microscopy study. *Brain Struct. Funct.* **2011**, *216*, 31–42, doi:10.1007/s00429-010-0295-4.

320. Dabir, D. V.; Robinson, M.B.; Swanson, E.; Zhang, B.; Trojanowski, J.Q.; Lee, V.M.Y.; Forman, M.S. Impaired glutamate transport in a mouse model of tau pathology in astrocytes. *J. Neurosci.* **2006**, *26*, 644–654, doi:10.1523/JNEUROSCI.3861-05.2006.

321. Gomez-Murcia, V.; Sandau, U.; Ferry, B.; Parrot, S.; Laurent, C.; Basquin, M.; Buée, L.; Boison, D.; Blum, D. Hyperexcitability and seizures in the THY-Tau22 mouse model of Tauopathy. *Neurobiol. Aging* **2020**, doi:10.1016/j.neurobiolaging.2020.06.004.

322. Choi, B.; Cho, W.; Kim, J.; Lee, H.J.; Chung, C.; Jeon, W.K. Increased expression of the receptor for advanced glycation end products in neurons and astrocytes in a triple transgenic mouse model of Alzheimer's disease. *Exp. Mol. Med.* **2014**, *46*, doi:10.1038/emm.2013.147.

323. Kulijewicz-Nawrot, M.; Verkhratsky, A.; Chvátal, A.; Syková, E.; Rodríguez, J.J. Astrocytic cytoskeletal atrophy in the medial prefrontal cortex of a triple transgenic mouse model of Alzheimer's disease. *J. Anat.* **2012**, *221*, 252–262, doi:10.1111/j.1469-7580.2012.01536.x.

324. Olabarria, M.; Noristani, H.N.; Verkhratsky, A.; Rodríguez, J.J. Age-dependent decrease in glutamine synthetase expression in the hippocampal astroglia of the triple transgenic Alzheimer's disease mouse model: Mechanism for deficient glutamatergic transmission? *Mol. Neurodegener.* **2011**, *6*, 1–9, doi:10.1186/1750-1326-6-55.

325. Le Douce, J.; Maugard, M.; Veran, J.; Matos, M.; Jégo, P.; Vigneron, P.A.; Faivre, E.; Toussay, X.; Vandenberghe, M.; Balbastre, Y.; et al. Impairment of Glycolysis-Derived L-Serine Production in Astrocytes Contributes to Cognitive Deficits in Alzheimer's Disease. *Cell Metab.* **2020**, *31*, 503-517.e8, doi:10.1016/j.cmet.2020.02.004.

326. Grolla, A.A.; Sim, J.A.; Lim, D.; Rodriguez, J.J.; Genazzani, A.A.; Verkhratsky, A. Amyloid-b and Alzheimer's disease type pathology differentially affects the calcium signalling toolkit in astrocytes from different brain regions. *Cell Death Dis.* **2013**, *4*, 1–7, doi:10.1038/cddis.2013.145.

327. Vanzulli, I.; Papanikolaou, M.; De La Rocha, I.C.; Pieropan, F.; Rivera, A.D.; Gomez-Nicola, D.; Verkhratsky, A.; Rodríguez, J.J.; Butt, A.M. Disruption of oligodendrocyte progenitor cells is an early sign of pathology in the triple transgenic mouse model of Alzheimer's disease. *Neurobiol. Aging* **2020**, *94*, 130–139, doi:10.1016/j.neurobiolaging.2020.05.016.

328. Wander, C.M.; Tseng, J.H.; Song, S.; Al Housseiny, H.A.; Tart, D.S.; Ajit, A.; Ian Shih, Y.Y.; Lobrovich, R.; Song, J.; Meeker, R.B.; et al. The Accumulation of Tau-Immunoreactive Hippocampal Granules and Corpora Amylacea Implicates Reactive Glia in Tau Pathogenesis during Aging. *iScience* **2020**, *23*, 101255, doi:10.1016/j.isci.2020.101255.

329. Adlimoghaddam, A.; Snow, W.M.; Stortz, G.; Perez, C.; Djordjevic, J.; Goertzen, A.L.; Ko, J.H.; Albensi, B.C. Regional hypometabolism in the 3xTg mouse model of Alzheimer's disease. *Neurobiol. Dis.* **2019**, *127*, 264–277, doi:10.1016/j.nbd.2019.03.008.

330. Edwards, M.M.; Rodríguez, J.J.; Gutierrez-Lanza, R.; Yates, J.; Verkhratsky, A.; Lutty, G.A. Retinal macroglia changes in a triple transgenic mouse model of Alzheimer's disease. *Exp. Eye Res.* **2014**, *127*, 252–260, doi:10.1016/j.exer.2014.08.006.

331. Rawat, V.; Wang, S.; Sima, J.; Bar, R.; Liraz, O.; Gundimeda, U.; Parekh, T.; Chan, J.; Johansson, J.O.; Tang, C.; et al. ApoE4 Alters ABCA1 Membrane Trafficking in Astrocytes. *J. Neurosci.* **2019**, *39*, 9611–9622, doi:10.1523/JNEUROSCI.1400-19.2019.

332. Larramona-Arcas, R.; González-Arias, C.; Perea, G.; Gutiérrez, A.; Vitorica, J.; García-Barrera, T.; Gómez-Ariza, J.L.; Pascua-Maestro, R.; Ganfornina, M.D.; Kara, E.; et al. Sex-dependent calcium hyperactivity due to lysosomal-related dysfunction in astrocytes from APOE4 versus APOE3 gene targeted replacement mice. *Mol. Neurodegener.* **2020**, *15*, 35, doi:10.1186/s13024-020-00382-8.

333. Zhang, L.; Xu, J.; Gao, J.; Chen, P.; Yin, M.; Zhao, W. Decreased immunoglobulin G in brain regions of elder female APOE4-TR mice accompany with Aβ accumulation. *Immun. Ageing* **2019**, *16*, 1–13, doi:10.1186/s12979-018-0142-7.

334. Li, G.; Bien-Ly, N.; Andrews-Zwilling, Y.; Xu, Q.; Bernardo, A.; Ring, K.; Halabisky, B.; Deng, C.; Mahley, R.W.; Huang, Y. GABAergic Interneuron Dysfunction Impairs Hippocampal Neurogenesis in Adult Apolipoprotein E4 Knockin Mice. *Cell Stem Cell* **2009**, *5*, 634–645, doi:10.1016/j.stem.2009.10.015.

335. Robertson, T.A.; Dutton, N.S.; Martins, R.N.; Taddei, K.; Papadimitriou, J.M. Comparison of astrocytic and myocytic metabolic dysregulation in apolipoprotein E deficient and human apolipoprotein E transgenic mice. *Neuroscience* **2000**, *98*, 353–359,

doi:10.1016/S0306-4522(00)00126-3.

336. Levi, O.; Lütjohann, D.; Devir, A.; Von Bergmann, K.; Hartmann, T.; Michaelson, D.M. Regulation of hippocampal cholesterol metabolism by apoE and environmental stimulation. *J. Neurochem.* **2005**, *95*, 987–997, doi:10.1111/j.1471-4159.2005.03441.x.

337. Brecht, W.J.; Harris, F.M.; Chang, S.; Tesseur, I.; Yu, G.Q.; Xu, Q.; Fish, J.D.; Wyss-Coray, T.; Buttini, M.; Mucke, L.; et al. Neuron-Specific Apolipoprotein E4 Proteolysis Is Associated with Increased Tau Phosphorylation in Brains of Transgenic Mice. *J. Neurosci.* **2004**, *24*, 2527–2534, doi:10.1523/JNEUROSCI.4315-03.2004.

338. Hartman, R.E.; Wozniak, D.F.; Nardi, A.; Olney, J.W.; Sartorius, L.; Holtzman, D.M. Behavioral phenotyping of GFAP-ApoE3 and -ApoE4 transgenic mice: ApoE4 mice show profound working memory impairments in the absence of Alzheimer's-like neuropathology. *Exp. Neurol.* **2001**, *170*, 326–344, doi:10.1006/exnr.2001.7715.

339. Tesseur, I.; Van Dorpe, J.; Bruynseels, K.; Bronfman, F.; Sciot, R.; Van Lommel, A.; Van Leuven, F. Prominent axonopathy and disruption of axonal transport in transgenic mice expressing human apolipoprotein E4 in neurons of brain and spinal cord. *Am. J. Pathol.* **2000**, *157*, 1495–1510, doi:10.1016/S0002-9440(10)64788-8.

340. Zhong, N.; Scearce-Levie, K.; Ramaswamy, G.; Weisgraber, K.H. Apolipoprotein E4 domain interaction: Synaptic and cognitive deficits in mice. *Alzheimer's Dement.* **2008**, *4*, 179–192, doi:10.1016/j.jalz.2008.01.006.

341. Graybeal, J.J.; Bozzelli, P.L.; Graybeal, L.L.; Groeber, C.M.; McKnight, P.E.; Cox, D.N.; Flinn, J.M. Human ApoE ε4 alters circadian rhythm activity, IL-1β, and GFAP in CRND8 mice. *J. Alzheimer's Dis.* **2015**, *43*, 823–834, doi:10.3233/JAD-132009.
